# Molecular, physiological and phylogenetic traits of *Lactococcus* 936-type phages from distinct dairy environments

**DOI:** 10.1038/s41598-018-30371-3

**Published:** 2018-08-22

**Authors:** M. Chmielewska-Jeznach, J. K. Bardowski, A. K. Szczepankowska

**Affiliations:** Institute of Biochemistry and Biophysics, Polish Academy of Sciences, Pawinskiego 5a, 02-106, Warsaw, Poland

## Abstract

Bacteriophage infection of *Lactococcus* species can cause serious disruption of dairy fermentation processes. The most common isolates from the dairy environment are *Siphoviridae* lytic 936-type phages. To gain specific knowledge about this group of phages in Polish dairies, we examined 90 isolates from 8 different locations. Based on restriction fragment length polymorphism analysis, coupled with physiological and molecular studies, the isolated phages were divided into 8 distinct groups. Whole-genome sequencing of single representatives from each phage group provided data about their biology and genetic composition. The phages present an overall conserved genome organization. High sequence homology to another Polish isolate, *Lactococcus* phage bIBB29, indicates their close phylogenetic relatedness to this strain. Such similarity may be suggestive of a general genome conservation among phages persisting in Polish dairies. Comparative genome analyses with other 936-type phages revealed several discriminative traits, including the presence and position of HNH endonuclease genes, varying number of *orfs* in the early gene region, and a putative TpeX gene. Interestingly, host range of the sequenced phages was restricted to *L*. *lactis* subsp. *lactis* biovar. *diacetylactis* strains. The results provide new data regarding phages present in the Polish dairy environment and permit analysis of their biology, genome composition and relatedness to other *Lactococcus* 936-type phages.

## Introduction

The majority of starter cultures used in the dairy industry for the production of sour cream, buttermilk, kefir or cheese contain, among other microorganisms, a mixture of *Lactococcus lactis* (*L*. *lactis*) strains. The role of starter bacteria is to promote fermentation, in which lactose is metabolized into lactic acid, with a simultaneous reduction of pH. In cheese production, approximately 1–10% of fermentations are slow or fail to produce lactic acid and do not reach the pH required for proper product formation^1^. It is estimated that 60–70% of technological disruptions encountered during industrial milk processing are caused by bacteriophage infections of sensitive lactococcal starter strains^2^. Despite various precautions, phage infections pose a real threat in milk factories^3–6^. The dairy environment serves as a constant source of phages, which may originate from raw milk, growth supplements, spontaneous prophage induction, factory equipment, or workers^7^. New phages can also arise in a factory through mutations and through recombination events between existing phages that infect the same sensitive host^8^. *Lactococcus*-infecting phages are among the most commonly isolated phages infecting any bacterial species^9^.

*L*. *lactis* phages belong to the order *Caudovirales* and have double-stranded DNA (dsDNA) genomes. According to the classification proposed by Deveau *et al*.^10^, lactococcal phages are grouped into ten distinct types based on nucleotide sequence homology. Eight of these phage types belong to the *Siphoviridae* family (with long, non-contractile tails and isometric or prolate heads), and the remaining two belong to *Podoviridae* (with short, non-contractile tails and isometric heads). Most lactococcal bacteriophages isolated from failed fermentations share morphological features with members of *Siphoviridae* and fall into one of the three most prevalent groups: 936, c2 and P335^11^. Additionally, the 936 and c2 types consist of only virulent bacteriophages, whereas the P335 group includes both virulent and temperate phages. The remaining *L*. *lactis* phage groups (1358, Q54, P087, 949, 1706, P034, and KSY1) are isolated less frequently, more often from raw milk than from failed fermentations^10^. As shown by numerous studies on dairy phage populations, 936-type species predominate in dairy environments, with a few exceptions^3,12–15^. This particularity is attributed to their high thermal resistance and ability to become air-borne and spread within the facility^3,4,16^. Time-based studies on the evolution of phages in a single dairy plant provide proof of the prevalence of 936-type phages in this environment over long periods of time^17^. Worldwide industrial and financial consequences of fermentation failures motivated studies on the dynamics of this specific bacteriophage community (its abundance, development, diversity, evolution, etc.). Various methods, including plaque assays, morphological characterization, host range, burst size, restriction fragment length polymorphism (RFLP) analysis, DNA-DNA hybridization, PCR-based approaches and, finally, the recently developed high-throughput sequencing, have been employed to characterize industrially emerging lactococcal phage isolates^8,10,12,18–24^. A better understanding of the bacteriophages that are prevalent in dairy plants is considered a crucial aspect of controlling phage infections in this environment. In an attempt to characterize 936-type phages collected during a 3-year period from 8 dairies located in various geographical regions in Poland, we initially screened 90 isolates for RFLPs. Based on distinct restriction patterns, phages were divided into 8 groups, and a representative from each group was subjected to whole-genome sequencing. The obtained data provide further information about the phages present in the milk environment, allowing the elucidation of their biology and genetic components and their comparison to already known 936-type phages that infect *Lactococcus* spp.

## Results

### RFLP profiling of phage isolates from whey samples

**Figure 1:**
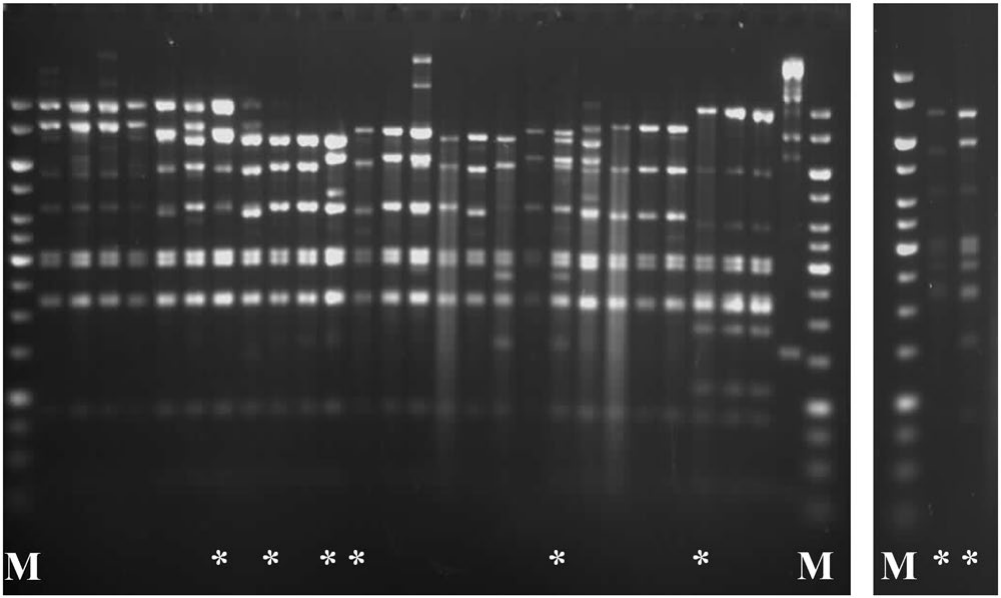
Restriction enzyme profiles of representative *Lactococcus* phages analysed in this study. DNA samples were cut overnight with *EcoRV* at 37 °C and treated with 50% formamide to dissociate *cos* ends prior to electrophoresis. M: 1-kb DNA Ladder (Fermentas). Unique phage DNA restriction patterns are marked by an asterisk (*).

Ninety phages isolated from disturbed fermentation samples from 8 factories were subjected to RFLP analysis, using chosen restriction endonucleases. In certain cases, the digested phage DNA samples varied only by a single band, making their discrimination rather difficult. The most differential and apparent DNA patterns were obtained by *EcoRV* digestion (Fig. 1). Through this analysis, 8 distinct phage groups with unique DNA restriction profiles were distinguished. Single representatives from each restriction group were subjected to whole-genome sequencing.

### Host range and infection dynamics

**Figure 2:**
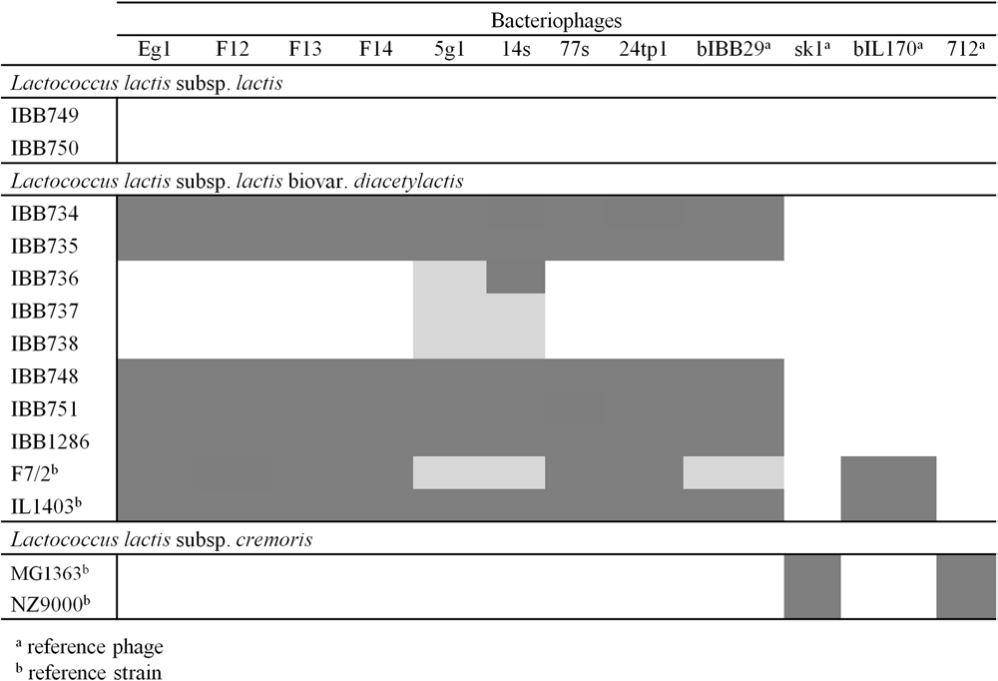
Host range of the studied phages tested on industrial *L*. *lactis* strains used at the time of whey sample collection. Grey-shaded boxes indicate clear plaques; light grey boxes indicate turbid plaques formed on the specified strains; white boxes signify lack of lysis. Phages bIBB29, sk1, bIL170 and 712 were used as references.

Phages selected for sequencing, representing distinct DNA restriction profiles, were examined for their host range against a set of 60 industrial, laboratory and environmental *L*. *lactis* isolates (Table 1). Of these, 11 strains were sensitive to all 8 phages, 4 strains exhibited selective sensitivity, and the remaining 75% of the strains were resistant to infection (Supplementary Table S1). Specifically, no detectable disturbance of bacterial cell growth was observed for any of the *L*. *lactis* subsp. *cremoris* or for any of the environmental *L*. *garvieae*, *L*. *plantarum*, *L*. *raffinolactis* or *L*. *laudensis* isolates. Moreover, none of the studied phages lysed *L*. *lactis* subsp. *lactis* strains other than biovar. *diacetylactis* (Fig. 2). In general, the examined phages exhibited highly similar host infection patterns. Most of them produced clear plaques on sensitive strains. The exceptions were phages 5g1 and 14s, which presented varying lysis efficiencies, repeatedly giving either turbid or clear plaques on selected strains. This property was the only distinguishing feature with respect to host range among the 8 sequenced phages. Notably, all sensitive strains except IL1403 and F7/2 (reference strains) were used by the dairy plants at the time of sample collection. In respect to those strains, the infection patterns of the phages were comparable to that of the bIBB29 bacteriophage, which was isolated previously from a Polish dairy plant, and differed from that determined for other 936-type phages tested (sk1, bIL170, 712), selected as references due to their noted distinct host ranges confined to *L*. *lactis* subsp. *lactis* (bIL170) or *L*. *lactis* subsp. *cremoris* (sk1 and 712)^25^. To gain further insight into the dynamics of infection, phage virulence was determined by one-step growth assays. The latent period of the phages was in the range of 15 ± 1 to 26 ± 3 min, indicating a fast infection rate. All phages were shown to complete the lytic cycle in 25 ± 1 to 34 ± 2 min (burst time), producing 43 ± 5 to 148 ± 6 progeny phage particles (burst size) (Table 2). No correlation between the three parameters of the phage lytic cycle was observed.

**Table 1:** Bacterial strains and phages used in study.

	Relevant features	Source/Reference
**Bacterial strains**		
IL1403	*L*. *lactis* subsp. *lactis* biovar. *diacetylactis* wild-type strain	^67^
F7/2	*L*. *lactis* subsp. *lactis* biovar. *diacetylactis*	^68^
MG1363	*L*. *lactis* subsp. *cremoris* wild-type strain	^69^
LM0230	*L*. *lactis* subsp. *cremoris* plasmid-free derivative of C2.	^70^
IBB477	*L*. *lactis* subsp. *cremoris* wild-type strain	^71^
NZ9000	*L*. *lactis* subsp. *cremoris* MG1363 derivative *pepN::nisRnisK* strain	MoBiTec®
IBB743, 749, 750, 752–754, 757–759, 761, 763, 764, 1784, 1796	*L*. *lactis* subsp. *lactis* industrial starter strains	Rhodia Food Biolacta
IBB734–738, 742, 744–748 751, 755, 756, 760, 762, 1286	*L*. *lactis* subsp. *lactis* biovar. *diacetylactis* industrial starter strains	Rhodia Food Biolacta
IBB694-700, 1788–1792	*L*. *lactis* subsp. *cremoris* industrial starter strains	Rhodia Food Biolacta
IBB338-342, 704	*L*. *lactis* subsp. *cremoris* natural isolates from traditional fermented milk products	IBB PAS collection
10LC	*L*. *lactis* natural isolate	courtesy of A. Rozga
3LC	*L*. *garvieae* natural isolate	courtesy of A. Rozga
76B	*L*. *plantarum* natural isolate	courtesy of A. Rozga
115	*L*. *laudensis* natural isolate	courtesy of A. Rozga
87B	*L*. *raffinolactis* natural isolate	courtesy of A. Rozga
Bacteriophages	Accession number*	
sk1	NC_001835.1	^72^
712	NC_008370.1	^73^
bIL170	NC_001909.1	^74^
c2	NC_001706.1	^75^
bIBBF14	MF346933	This study
bIBB5g1	MG253647	“
bIBB14s	MG253648	“
bIBB24tp1	MG253649	“
bIBB77s	MG253650	“
bIBBEg1	MG253651	“
bIBBF12	MG253652	“
bIBBF13	MG253653	“

*Complete genomic sequences of *Lactococcus* phages analyzed in this study were deposited in GenBank under the given accession numbers.

**Table 2:** Burst size, burst time and latent period based on one-step growth assay of the studied phages.

Phage	Burst size [no. of particles]	Latent period [min]	Burst period [min]
5g1	43 ± 5	20 ± 0	29 ± 1
14s	54 ± 4	28 ± 3	34 ± 2
24tp1	54 ± 2	18 ± 0	29 ± 1
F12	68 ± 1	20 ± 0	31 ± 1
F14	81 ± 3	20 ± 0	31 ± 0
Eg1	95 ± 4	20 ± 0	29 ± 1
F13	135 ± 5	20 ± 0	29 ± 0
77s	148 ± 6	15 ± 1	25 ± 1

Values are the means of three determinations ± standard deviations.

### Examination of phage morphology

**Figure 3:**
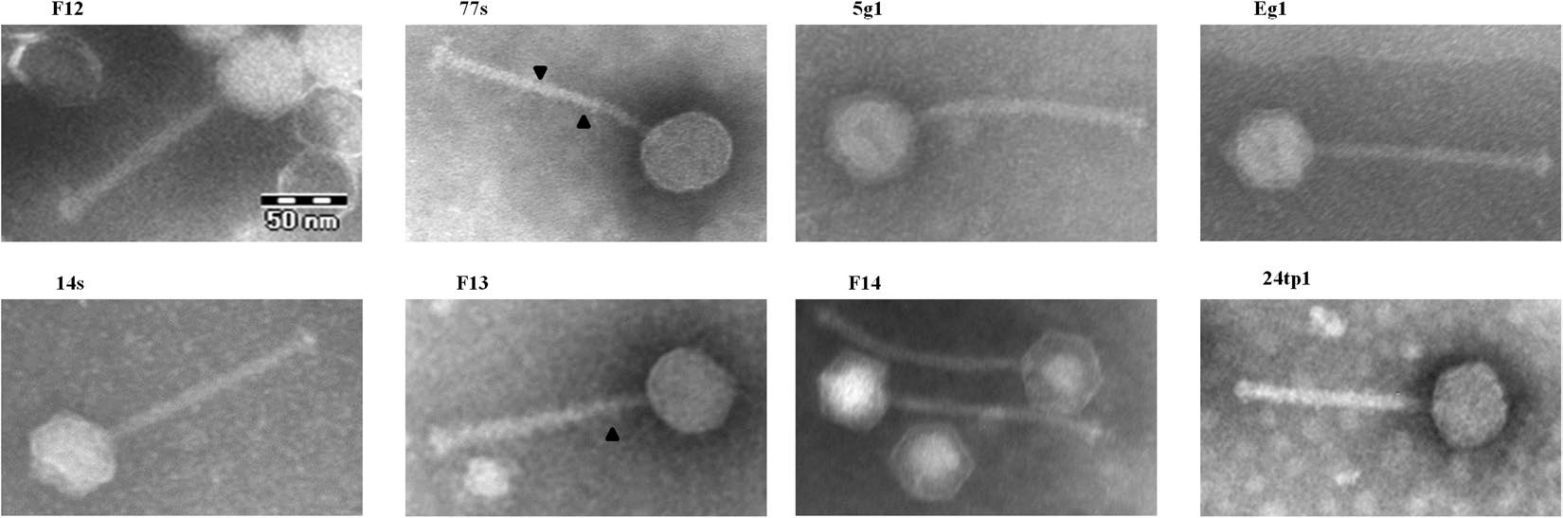
Electron micrographs of *Lactococcus* phages sequenced in this study. Arrowheads point to potential spiral-like structures around the phage tails.

Examination of the sequenced phages by electron microscopy confirmed their typical *Siphoviridae* morphology (Fig. 3). All 8 phages were shown to possess small isometric heads and long tails. Similar to other 936-type isolates^26^, a large disc-like structure (baseplate) was observed at the tip of the phage tails. On several electrographs, potential spiral-like fibres/cross-striations are visible. Conversely, collar-like structures (or neck passage structures; NPS) were not observed. The results of the electron microscopy analyses are summarized in Table 3.

**Table 3:** Characteristic morphological features of the studied phages.

Phage	Electron microscopy			Plaque diameter* [mm]
	Tail diam. [nm]*	Head diam. [nm]*	Additional structures	
5g1	127 × 8	50	nd	3.6
Eg1	143 × 12	58	nd	4.2
F12	132 × 10	55	nd	3.8
F13	135 × 11	55	spiral-like structures	3.7
F14	131 × 9	57	nd	3.6
14s	145 × 12	65	nd	4.0
24tp1	147 × 12	66	nd	2.9
77s	137 × 10	55	spiral-like structures	4.0

The size of phage particles was calculated using measure IT 5.0 (Olympus Soft Imaging Solutions GmbH) software.

*Mean from n = 5 phages.

nd – none detected.

### Phage classification by multiplex PCR

**Figure 4:**
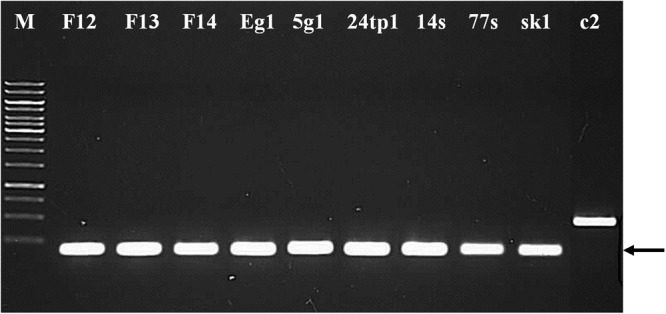
Phage typing using multiplex PCR. Unique fragments of phage genomes were amplified using a mix of primers complementary to the conserved genomic regions of 936- and c2-type phages. Arrows indicate the 936-type-specific band of 179 bp. Lane 1: 1-kb DNA Ladder (Fermentas), lanes 2–9: DNA fragments generated from respective phage lysates, lane 10: DNA fragment generated from phage sk1 lysate as a positive control for 936-type, lane 11: DNA fragment generated from phage c2 lysate as a negative control for 936-type.

Phage typing using a PCR-based method allowed the classification of the sequenced phages as 936-type. A specific 179-bp DNA fragment was detected for the 8 phages under study, as well as for sk1 (positive control, 936-type phage), but not for *Lactococcus* phage c2 (negative control, c2-type phage) (Fig. 4). These findings corroborate those of the electron microscopy studies and confirm that the examined phages belong to the 936 type.

### General features of *Lactococcus* phage genomes

**Figure 5:**
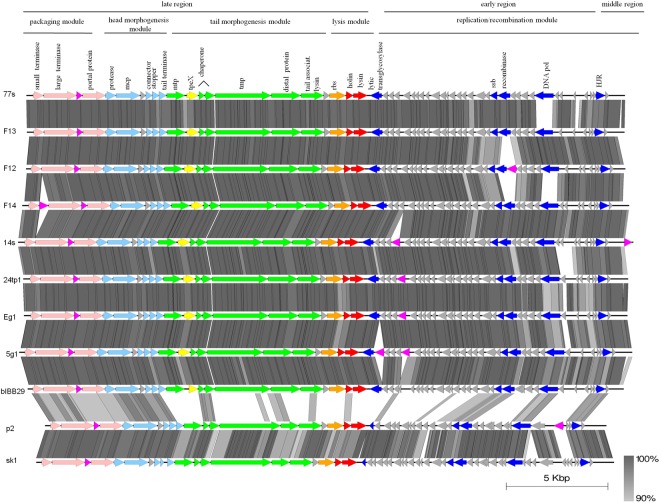
Schematic comparison of the *Lactococcus* phage genomes sequenced in this study. Arrows represent predicted genes and are coloured according to their putative function (indicated above each arrow). Genes encoding packaging proteins are in light pink; head morphogenesis, light blue; tail morphogenesis, green; TpeX-encoding genes, yellow, RBP-encoding genes, orange; host cell lysis, red; *orfs* of unknown function, grey; HNHE-encoding genes, pink; annotated functions of the early and middle genome region are in navy blue. The genomes of phages bIBB29, p2 and sk1 are shown as references. The grey shading between genomes represents tblastx matches according to the BLAST identity scale (%).

**Figure 6:**
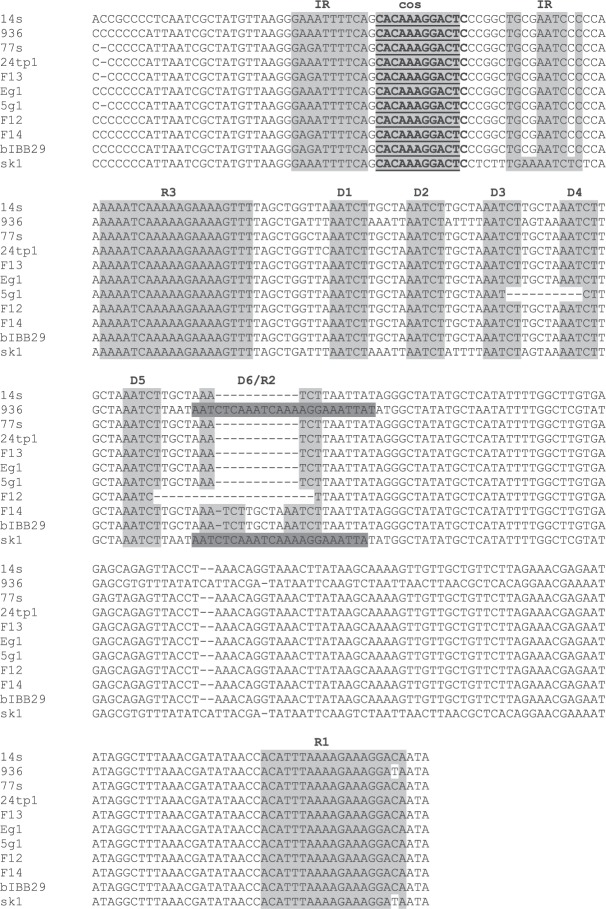
Comparative sequence analysis of aligned *cos* regions of *Lactococcus* phages sequenced in this study using the ClustalOmega tool. Sequences characteristic of phage 936-type *cos* regions are shaded in grey. *Cos* sites are underlined. IR: inverted repeats, R1-3: putative terminase binding sites, D1-6: direct repeats.

Single representatives of *Lactococcus* 936-type phages representing the 8 unique DNA restriction profiles determined in this study were subjected to whole-genome sequencing. The sequenced genomes were found to consist of linear dsDNA, 29.3–30.2 kb in size, and possess a G-C content (34.6–34.9%) similar to that reported for *Lactococcus* spp. (Table 4). An overall conserved genetic organization, with three transcription regions comprising early, middle and late genes, was identified in all phages sequenced in this study and was consistent with the genome organization of other known 936-type phages^27,28^. The identified open reading frames (*orf*s) within each region were determined to be encoded on the same strand and oriented in the same direction (Fig. 5). No tRNA or tmRNA encoding sequences were detected on either strand. In total, 53–57 *orf*s were identified. Approximately 40% of them were assigned putative functions based on homology of respective products to proteins currently present in databases. The genomes of the sequenced phages were determined to possess 11-nt cohesive ends (*cos*) at their extremities, between the middle- and late-expressed genes. The identified *cos* sites were found to be conserved and identical in sequence to those of other 936-type phages (CACAAAGGACT)^28,29^. The surrounding region was established to contain typical features described previously for 936-type *cos* sites: 10-bp inverted repeats on each side of the *cos* sites, 5–6 direct repeats (AATCT) and two complete sequences corresponding to two of the four previously identified putative terminase binding sites (R1 and R3)^30^. Sequence alignment showed that one of the direct repeats (D6) present in 7 out of 8 studied phages (F14) was a truncated R2 terminase binding site (Fig. 6).

**Table 4:** Main characteristics of the studied phages based on sequencing data.

Phage	Dairy plant	Genome length (bp)	GC content (%)	No. of predicted genes	No. of ORFs with predicted functions
Eg1	1–5	29,659	34.7	54	21
5g1	2, 4	30,132	34.6	55	22
24tp1	3	29,736	34.8	55	21
F12	6	29,454	34.9	55	21
F13	6	29,374	34.8	53	20
F14	6	29,837	34.8	56	21
14s	7	30,249	34.6	57	22
77s	8	29,412	34.8	54	20

Based on the arrangement of the phage genomes and functional annotation of predicted *orf*s, 4 regions could be identified: (i) DNA replication/recombination in the early- and middle-expressed region and (ii) lysis, (iii) head and tail morphogenesis, and (iv) DNA packaging modules in the late-expressed region (Fig. 5). In general, Mauve processive alignment revealed significant sequence homology and strong conservation of gene synteny between the genomes of all 8 phages examined in this study (data not shown). However, subsequent in-depth sequence analyses using bioinformatic tools allowed the mapping of genomic regions of distinct divergence.

#### DNA replication/recombination region

Among the 31–36 *orfs* identified in the DNA replication/recombination region of the sequenced phages, only 5–7 have been assigned putative functions based on significant similarities to entries found in public databases (Fig. 5). These included a putative single-strand DNA annealing protein (SSAP), single-strand DNA binding protein (SSB), DNA polymerase subunit, lysozyme-like superfamily protein, HNH homing endonuclease (HNHE) and Holliday junction resolvase (HJR). SSAP proteins play an essential role in recombination and proteins from the SSB family are known to play an accessory function in the process^31,32^. The putative SSAPs and SSBs of the sequenced phages were all identical, except for phage F12, and exhibited homology (100% identity) respectively to GP42 and GP41 of *Lactococcus* phage bIBB29, which was isolated previously from a different Polish dairy plant^29^. For phage F12, the putative SSAP (GP41_F12_) contained short sequence deletions in the N-terminal and central region, and more closely resembled the SAK protein of *Lactococcus* phage Phi10.5 (97% identity vs. 58% identity to GP42_bIBB29_). In turn, the hypothetical SSB protein of phage F12 (GP40_F12_) contained single amino acid substitutions throughout the central and C-terminal part, compared to the remaining phages, and was most homologous (99% identity) to the putative SSB of *Lactococcus* phage Phi19.3. Interestingly, an *orf* identified just upstream of *gp41*_F12_ was determined to encode a putative HNHE protein (GP42_F12_). The BLASTp search showed high sequence homology of GP42_F12_ to the NUMOD4 motif found in *Chlamydia trachomatis* (92% identity). The presence of a putative HNHE could indicate a possible horizontal acquisition of the adjacent DNA regions, including *gp40-gp41*. The third recognized function in the early-expressed gene region was DNA polymerase. In all 8 phages sequenced, this hypothetical protein best matched (97–100% identity) the putative DNA polymerase subunit of *Lactococcus* phage bIBB29 (GP47_bIBB29_), with single amino acid differences (Supplementary Fig. S1). The exception was in phages F12 and F13, for which the predicted DNA pol exhibited high sequence divergence (45% identity) with respect to bIBB29, most closely resembling the putative DNA polymerase subunit of *Lactococcus* phages R3.4 and P680 (98% identity). A distinct feature of the early-expressed region among the 8 sequenced phages was the presence and location of putative HNH homing endonuclease genes (Fig. 5). Apart from F12, phages 14s, 24tp1, and Eg1 were also determined to possess one putative HNHE, and phage 5g1 was determined to possess two such endonucleases (GP23 and GP27). In contrast, phages 77s, F13 and F14 lacked a gene with such function in this particular genomic region. For phage 5g1, the putative protein products encoded by *gp23* and *gp27* exhibited the highest sequence homology to a predicted HNH endonuclease from *Staphylococcus fleurettii* (locus tag: B2G86_12885) (99% identity) and GP23 of *Lactococcus* phage 936 (96% identity), respectively. The three remaining phages (14s, 24tp1 and Eg1) encoded an HNH endonuclease identical to GP27_5g1._ The HNH endonucleases predicted in the early-expressed gene region of the sequenced phages possessed a conserved zinc-binding loop domain also found in the HNHc superfamily. Proteins from this group are similar to the F-TflII endodeoxyribonuclease of *E*. *coli* T5 phage, described to act on asymmetric DNA substrates (coding or template) to introduce single-strand breaks^33^. HHPRED analysis allowed the determination of structural homology of the identified HNHEs to the catalytic motif of *Bacillus* phage SPO1 HNH, I-HmuI (E-values: 1.6e-30 for GP23_5g1_ and 4.8e-27 for the remaining products). The last *orf* of the early-expressed genes, directly neighbouring the late-expressed gene region, was found to encode a putative lysozyme-like superfamily protein containing a membrane-bound lytic murein transglycosylase MltE domain (COG0741). Lytic murein transglycosylases are involved in the cleavage of the β-1,4 glycosidic bond between the N-acetylglucosaminyl and N-actetylmuramoyl residues of the bacterial peptidoglycan layer^34^. Based on studies in the phages T7, PRD1 and P1, lytic transglycosylases were suggested to play a role during the infection of host cells, possibly due to the localized destabilization of the bacterial cell wall peptidoglycan layer^35^. The putative lytic transglycosylases of the phages sequenced in this study exhibited a high sequence similarity to the GP22 protein of phage bIBB29 (98–100% identity). At the structural level, the identified products resembled the *E*. *coli* EtgA muramidase (E-values: 2.9e-8 to 6.4e-10, depending on the phage). The remaining *orfs* in the early-expressed gene region could not be assigned functions using the BLASTp, HHPRED or HMMER tools but exhibited high homology (>90% identity) to proteins encoded in the same genomic positions by *Lactococcus* phage bIBB29, with some exceptions (Fig. 5). The middle-expressed gene region of 936-type phages is also postulated to encode proteins engaged in phage DNA replication/recombination events. For all phages sequenced in this study, this region was found to contain 4 small *orfs*, similar to the case in other 936-type phages^28^. Among them, the largest (483 bp), mid-*orf* 3, was determined to encode a putative Holliday junction resolvase (HJR) with a RuvC domain, which exhibited significant homology (99% identity) to HJRs of several *Lactococcus* phages, including ASCC281, ASCC284, ASCC310, ASCC358 and ASCC365. Notably, in phage 14s a putative HNHE function was identified for the protein encoded by the last *orf* of the middle gene cluster. The hypothetical protein product exhibited high sequence identity (99%) to the HNH endonuclease of the *Lactococcus* phage P113G, which has a conserved HNHc superfamily zinc-binding loop domain. Similar to the case for the other HNHEs identified in the early gene regions, the best structural match for this protein was the HNH catalytic motif of *Bacillus* phage SPO1 I-HmuI processing endonuclease (E-value 1e-21). Rest of the products encoded in the middle-expressed region remain unannotated; no relevant sequence or structural homology to proteins existing in databases was noted.

In 936-type phages, the late-expressed region encodes products engaged in lysis, head and tail morphogenesis and packaging. Comparative genome analysis of this region among the studied phages showed an overall high sequence conservation (>90% identity). The late regions have identical genetic organization in all 8 phages except phage F14 (Fig. 5). This gene arrangement is distinct from that of other 936-type phages^28^, differing essentially in the presence or lack of a putative HNHE gene between the small and large terminase subunits. In contrast, the organization of the phage F14 late region resembles that reported earlier for phage Phi5.12^28^.

#### Lysis module

The lysis module of the phage late region directly neighbours the early-expressed gene region and encodes two putative proteins, holin and lysin, that play essential roles in the liberation of phage progeny from host cells. BLASTp analysis classified the endolysin encoded by the studied phages to N-acetylmuramoyl-L-alanine amidases, which cleave the amide bond between two moieties and thus liberate progeny phage particles from host cells^36^. The identified hypothetical holin proteins were shown to contain 3 transmembrane domains, classifying them as class I holins^37^ (Supplementary Fig. S2).

#### Head and tail morphogenesis module

The morphogenesis region, located upstream of the lysis module, carries genes encoding structural proteins that are engaged in head and tail formation and host infection. Based on analogies of position and *orf* size, coupled with high sequence homology (≥94% identity) to the corresponding gene products of *Lactococcus* phages bIBB29 and p2, hypothetical functions were assigned to the putative *orfs* within the morphogenesis modules of phages sequenced in this study (refer to Fig. 5 for details). Among the important proteins encoded in this region is the RBP, which is essential in phage–host interactions as it recognizes receptors (carbohydrate residues) on the bacterial cell surface and plays a crucial role in the attachment of the virion to the host cell^38^. Divergences in RBP sequence (mainly in the C terminus) have been linked with variations in host range^39,40^. Comparative analysis of the putative RBPs of the sequenced phages revealed their 100% sequence identity, except for phages 77s, 24tp1 and 5g1, which carried a single amino acid substitution. Multiple sequence alignment with representative proteins of the currently identified RBP groups in 936-type phages^41^ showed high conservation in the N-terminal part and a variable C terminus, which was consistent with previous reports (Supplementary Fig. S3)^39,40,42^. The highest similarity, spanning the whole sequence, was found for the putative RBP of phage bIBB29 (GP19) (99–100% identity) and, to a lesser extent, *Lactococcus* phages P475 (97% identity) and 645 (94% identity). Phylogenetic analysis showed clustering of the putative products to group III RBPs, confirming their significant homology (Supplementary Fig. S4). Previous studies of 936-type phage genomes reported that the greatest sequence variations within the late-expressed gene region occur in the surrounding of genes encoding the major capsid and tail proteins (MCP and MTP), the tape measure protein (TMP) and the receptor binding protein (RBP)^28,43^. For phages sequenced in this study, the greatest divergence in the region was detected within the *orf* localized just downstream of the putative *mtp*. A search for homologues using BLASTp identified the 151-aa putative tail structural protein of *Lactococcus* phage bIBB29 (GP12_bIBB29_) as the best match (99–100% identity), but with only 76–78% sequence coverage. However, manual correction of the start position of bIBB29 *gp12* to match that automatically detected for the corresponding *orf* of phage 77s increased the coverage value to 96–100% and identity ≥98%. Further scrupulous inspection of the size and position of this uncharacterized *orf* in respect to the morphogenesis module of other 936-type phages, indicated the presence of a potential tail protein extension (*tpeX*) gene. Multiple sequence alignment with putative TpeX sequences identified previously in other 936-type phages (i.e., Phi93, Phi145) showed homology (65% identity) in the C-terminal part (Supplementary Fig. S5). In earlier studies, TpeX was described to contribute to the formation of an alternative elongated product, major tail protein extension, as the result of a +1 translational frameshift at the end of the *mtp* gene sequence. To determine whether this is also the case for phages sequenced in this study, the intergenic region between the *mtp* and the downstream putative TpeX-encoding *orf* was examined for signals of a possible translational shift. The analysis revealed a CCC.UAG sequence at the end of the *mtp* gene, which was previously described as the site of a ‘shifty stop’ for several other 936-type phages (e.g., PhiE1127, PhiB1127, Phi5.2, Phi4.2, PhiCO139)^28^. Bioinformatic simulation of the +1 translational frameshift in this DNA region for phages sequenced in this study (CCC.UAG → CCC.UAG) permitted the formation of largely identical (with single amino acid differences) putative protein products (520 aa), which best matched the major TpeX of *Lactococcus* phage Phi5.12 (68–69% identity, 99% coverage) and Phi 4.2 (65% identity, 100% coverage). Close clustering of these putative protein products was confirmed by maximum-likelihood phylogenetic analysis (Supplementary Fig. S6). Notably, HHPRED analysis indicated that the C-terminal part exhibited high structural homology (E-values: 1.1e-34 to 6.7e-35) to the minor structural protein 5 (5E7T_B) of *Lactococcus* phage TUC2009 (P335 species), described to be a component of the baseplate structure that is involved in binding to host receptors^44,45^. It is plausible that these proteins could play a role in the phage–host interactions of the newly sequenced phages. The presence of the MTP extension protein was found to manifest as spiral-like structures along the tail that were visible under an electron microscope. Similar structures were also observed in several of the phages under study (e.g., 77s, F13) (Fig. 3). However, both the +1 frameshift and the formation of a larger protein, as well as the localization of the protein (in spiral-like forms or in baseplate structures), require further experimental investigation.

#### Packaging module

The packaging module is located upstream of the morphogenesis region and contains genes encoding the portal protein and the small and large terminase units. Together, they form the DNA packaging complex that recognizes and cuts phage DNA concatemers into properly sized genome fragments, which subsequently pass through a hole formed by the portal protein into the empty phage capsid^46^. The recognized putative small and large terminase units and the portal protein are identical among the studied phages and exhibit the highest sequence homology (100% identity) to corresponding proteins encoded by *Lactococcus* phage bIBB29. One exception is the predicted large terminase subunit of phage F14, which best matches proteins from other lactococcal phages, including bIL170 (99% identity). The packaging module of 936-type phages may carry additional genes, such as those encoding homing endonucleases or methyltransferases (MTases)^28^, which are often positioned between the genes encoding the large and small terminase units. Among the studied phages, only F14 was found to encode a putative HNH endonuclease in this position (with a partial HNH_3 domain in the N terminus and HNH_ASNC domain in the C-terminal part). The predicted product presented high structural similarity (E-value 6.8e-27) to the HNH catalytic motif of *Bacillus* phage SPO1 I-HmuI. Additionally, all 8 phages encoded a highly conserved protein with putative HNH activity (including an HNHc family domain) directly upstream of the portal function. Interestingly, the two best matches of this sequence (100% identity) were the putative HNH endonucleases of *Lactococcus* phage bIBB29 (GP03) and of the gram-negative bacterium *Porphyromonas macacae* (locus tag: HR11_02520). Moreover, strong structural resemblance (E-value 3.7e-12) to the HNH protein of thermophilic phage E2 of *Geobacillus* sp. (5H0M) was noted.

### Genome-based phylogeny of the sequenced phages

**Figure 7:**
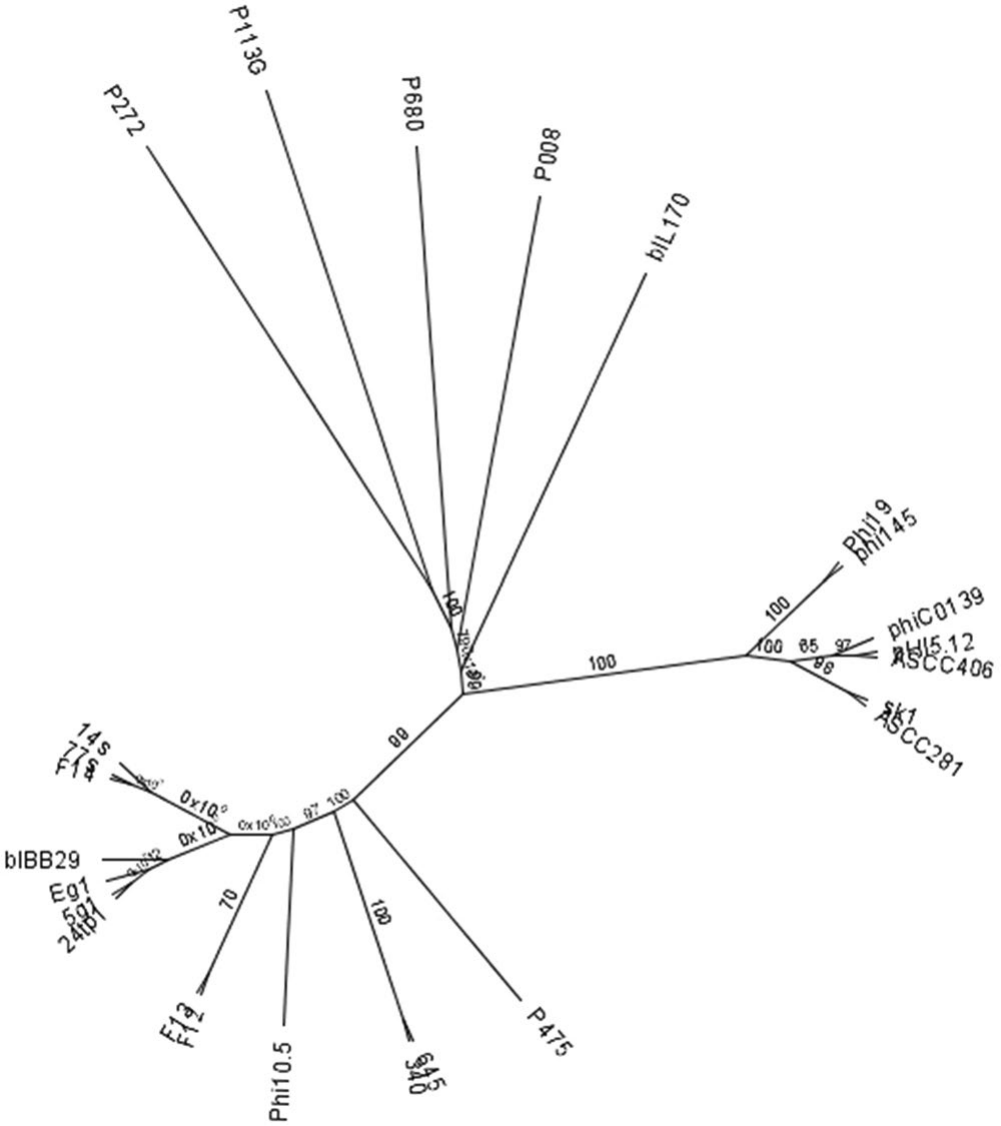
Maximum-likelihood phylogenetic tree of *Lactococcus* phages sequenced in this study. Phage phylogenies were reconstructed from multiple alignment of 936-type phage whole genome sequences generated using MUSCLE with 8 iterations (default settings). A maximum-likelihood tree was constructed using PHYML and the Junker-Castor substitution model with 100 bootstrap resamplings. Consensus support (%) is marked at each node.

To analyse the genetic relationship between the sequenced phages and other 936-type phages, an unrooted phylogenetic tree was built using the maximum-likelihood (ML) method. For comparison, we chose representative 936-type phages, which based on multiple genome alignments were previously reported to form distinct clades^28^. The obtained results show clustering of the phages examined in this work, which confirms their high genome similarity (Fig. 7). Albeit closely associated, the 8 phages are shown to be divided into two branches and additional sub-branches. Moreover, a clear association with bIBB29 was noted. Conversely, the ML tree indicates a separation of the studied phages from other 936-type phages, including those previously assigned to the same cluster with bIBB29^28^. Interestingly, several of the sequenced phages that were isolated from different geographical locations (Table 4) are shown to cluster closely together (e.g., 5g1 with 24tp1 and F14 with 77s and 14s). Conversely, phages obtained from the same location at the same time point (F12, F13, F14) were observed on different sub-branches of the phylogenetic tree. Based on comparative analyses of whole-genome sequencing data obtained in this study, the highest level of phage diversity was assigned to the early gene regions. Dot plot analyses confirmed the observed genome-wide sequence homology between all 8 phages (data not shown). Stretches of disturbed nucleotide synteny were mapped to three parts of the early gene region (upstream of the putative DNA pol encoding gene, directly downstream of the hypothetical *ssb* gene and approximately 1 kb upstream of the potential lytic transglycosylase gene) and to several sites of the late region. More generally, of the 29 genes reported by Murphy *et al*.^28^ to constitute the core genome of 936-type phages, half of them were identical between the studied phages. Despite the high homology of the genome sequences, several features (described earlier) were recognized as differentiating factors among the studied phages. These included (i) the varying number of *orf*s upstream of the putative DNA polymerase gene and (ii) the presence and position of *orfs* encoding putative HNHEs. With regards to the latter, only phage pairs Eg1 and 24tp1 and 77s and F13 exhibited the same genetic organization, encoding either one or no HNHE homologues, respectively. The distinct identity of these phages was determined by running BLASTn analysis on their genome sequences, which revealed high, but not identical, sequence similarity (for 24tp1 and Eg1: 99% identity with 99% coverage, for 77s and F13: 97% identity and 99% coverage). The close clustering of the studied phages indicates strong conservation of phage DNA sequences (>90% identity) in the dairy environment. This finding is quite intriguing, given that the phages sequenced in this study were isolated at varying points in time from different dairy plants and geographical regions across Poland.

## Discussion

Phage contamination of dairy factories remains one of the main causes of fermentation failures. Infections of *L*. *lactis* strains, used mainly in the production of cheese, buttermilk and sour cream, constitute the largest part of the technical problems experienced in this field. The majority of phages infecting the *L*. *lactis* species in milk plants identified thus far are 936-type phages^8^ (and references within). The persistence of 936-type phages in this environment is due at least in part to their high thermal resistance and volatility and the use of phage-sensitive starter strains^47^. The characterization of lactococcal phages in terms of their morphological and molecular features, and more recently, their genetic content, is regarded as a key step in understanding phage persistence and exploring the means to reduce their presence in milk plants. Our study focused on industrially prevalent phages isolated from 8 different dairies across Poland during a 3-year period. The occurrence and diversity of phages in dairy plants from various geographical locations have been analysed in a number of works^10,17,21,22,24^. The common features of industrial phage infection kinetics are short latent periods and relatively high burst sizes^48^. The results of our one-step growth assays are consistent with this observation. Previous studies point to the importance of constant screening of phage populations to minimize their impact on fermentation processes and to permit the use of appropriate strain rotation strategies. While host range and DNA RFLP analyses are the principal techniques used in phage typing, phage characterization through whole-genome sequencing is far more valuable for providing information on newly emerged phages and for understanding the population dynamics of phages in the dairy environment^8,9,28,49^. In this work, we sequenced 8 distinct phages identified among 90 isolates derived from Polish dairies. Comparative analysis of resultant phage genome sequences revealed several interesting traits. The most noticeable feature is the low diversity among the studied phages, regardless of the region or time of isolation. This finding is quite intriguing; however, several other studies have also reported the identification of highly homologous 936-type phages isolated from geographically separated regions. A study by Moineau *et al*.^13^ showed that 936-type phages isolated from buttermilk factories in over 20 US states presented similar DNA restriction patterns. This feature seemed to be strain independent, as different starter cultures were employed in each dairy plant. Another study by Raiski & Belyasova^22^ noted a widespread distribution of 936-type phages presenting similar restriction profiles. The same work showed that phages with identical DNA digestion patterns may infect different hosts. This implies a lack of strong correlation between host range and RFLP profile. Our observations seem to be in line with this hypothesis–all 8 phages essentially infected the same starter strains, yet varied in their DNA restriction patterns. This clearly shows that the determination of relationships among phages based on this factor alone may be largely misleading. In this regard, the generally accessible whole-genome sequencing allows a direct examination of phage genomic content and supplies far more informative and detailed data on phage biology and relationships. The close relatedness of the 8 phages determined by whole-genome sequencing data correlates with host range. The infection resistance of lactococcal strains from natural environments (raw milk or plant material) corroborates the narrow activity range of the studied phages. The host infection patterns of the phages were restricted to biovar. *diacetylactis* starter strains used by milk plants at the time of sample collection (except two reference strains – IL1403 and F7/2), which suggest high selective adaptation to dairy settings. The only other phage that could produce plaques on the same set of industrial strains was bIBB29, which also derived from a Polish dairy plant^28^. Although the time and place of isolation differed for bIBB29 and the phages sequenced in this study, our phylogeny analysis showed them to cluster together. Moreover, the argument that homologous phages lyse the same sensitive strain more readily than phages with lower sequence identity^14^ seems also to apply in our case, as the other reference phages (i.e., sk1, bIL170, 712) were ineffective on the same strains. The main phage factor determining the host range specificity is the RBP, particularly the C-terminal part of the protein, which is engaged in interaction with the host cell surface^39,40^. The phages examined in this work exhibited significant homology among their putative RBPs. Phylogeny analyses revealed their close clustering with group III RBPs including phage bIBB29 (≥90% identity)^28^. Both results substantiate the identical infection patterns of bIBB29 and the phages sequenced in this study. In contrast, host range variations were noted for sk1, bIL170 and 712. Based on host range and differences in the RBP C terminus, these three reference phages were previously determined to represent different phage subgroups^25,39^ and infect either *L*. *lactis* subsp. *cremoris* (sk1 and 712) or *L*. *lactis* subsp. *lactis* (bIL170); although since then additional subclasses of 936-type phages have been reported^28,43^. Only bIL170 was able to produce turbid plaques on biovar. *diacetylactis* F7/2 strain. Notably, the RBP of bIL170 was the most homologous in its C-terminal part to the corresponding proteins of the 8 phages examined in this study. Variations in RBP sequences do not necessarily have to be the sole factors responsible for discrepancies in host range. Indeed, phages with almost identical RBPs were reported to infect different bacterial strains^29^. Recent studies postulate the engagement of other factors, such as the neck protein structure (NPS) or tail protein extension (TpeX), in infection^28,43^. However, while the RBP gene is always present in genomes of 936-type phages, the two aforementioned proteins are apparently inessential. Their role in host tropism is postulated to be secondary, and they are thought to potentially engage in host range extension^28^. The phages analysed in our study seem not to possess a gene encoding the NPS homologue, which was described based on electron micrographs as forming a single or double disc between the head and the tail^24,28,29^. Instead, all of the phages encode a highly conserved hypothetical TpeX, previously described to account for characteristic spiral-like structures around the phage tail. The identification of a possible +1 frameshift at the end of the *mtp* gene located directly upstream of the putative *tpeX* suggests a possible fusion of the two respective proteins. We also identified the same, previously unreported, sequence characteristic of a ‘shifty stop’ in the genome of phage bIBB29 at the end of *gp11* (data not shown). We hypothesize that this determinant allows the formation of an extended 520-aa GP11-GP12 product. The significance of this genetic determinant is being evaluated; however, it can be speculated that the formation and sequence of an extended MTP protein may contribute to the host infection patterns and the dominance of these phages under specific conditions, such as those of the dairy environment. Arguments for this notion can be found when examining putative TpeX sequences of phages possessing group III RBPs (645, 340, P475, bIBB29 and phages examined in this study). Compared with the 8 sequenced phages, the TpeX protein of phage bIBB29 is highly homologous (≥98% identity), while that of phage P475 exhibits low (≥37%) identity. In turn, phages 645 and 340 seem to lack the *tpeX-*encoding gene. It is plausible that sequence variations in TpeX can account also for the discrepancies in infection patterns among phages representing the same RBP group. Evidence for this hypothesis are supplied by previous studies, in which phages 645 and P475 were shown to exhibit a limited host range towards *L*. *lactis* subsp. *lactis* biovar. *diacetylactis* strains in comparison to bIBB29^29,50^. This observation contradicts the results obtained by Mahony *et al*.^43^, in which phages 645 and P475 were shown to successfully infect the same biovar. *diacetylactis* strains tested earlier. An explanation for this may be that formation of MTP extension occurs only under specific conditions. Determination whether +1 frameshift demands specific factors or is an incidental process will need further studies. Only detailed mutational analyses may fully elucidate the role of TpeX in host tropism. Other potential determinants that may contribute to the dominance of phages in a specific setting are HNH homing endonucleases. Arguments for this can be found in a study by Goodrich-Blair & Shub^51^ involving the I-HmuI of *B*. *subtilis* phage SPO1, one of the structural homologues identified for the HNHEs of the currently sequenced phages. The results of this work showed that I-HmuI has higher preference for heterologous phage DNA, which may be especially advantageous during co-infection. Whether this also applies for the HNHEs of *Lactococcus* 936-type phages has yet to be determined. The overall high similarity among the sequenced phages and bIBB29 may be suggestive of a general genome conservation among the Polish dairy phages. Also previous studies report a high homology among dairy phages isolated from other countries^28^. To determine whether this holds true also in Poland, further studies on a larger group of phages are required. The close relatedness of the sequenced phages could signify their quite recent evolution, most likely due to point mutations or recombination events that took place within the host. It may be that the analysed phages derived from a single, common ancestral phage, which was carried over to and evolved differentially in each dairy plant. A comprehensive study by Castro-Nallar *et al*.^49^ posited that phages isolated from dairy plants are most likely disseminated in the environment and between individual factories by human carriers. Other previously reported potential sources of contamination, such as milk powder or whey protein concentrates, cannot be ruled out^7^. The growing number of sequenced 936-type phage genomes allows comparative analyses of their genetic content and the identification of variable regions. The current study is the first to examine and compare a set of genomes of 936-type phages isolated from the Polish dairy settings. The obtained results bring us closer to understanding the basis of the prevalence of 936-type phages in specific environments and facilitate the search for host range determinants. In conclusion, our work shows the significance of whole-genome data collected from industrially isolated phages in providing valuable insights for applied microbiology and the development of strategies for combating phage infections in dairy environments.

## Materials and Methods

### Bacterial strains and growth conditions

The bacterial strains and phages used in this study are listed in Table 1. *L*. *lactis* strains were grown in M17 broth (Oxoid) supplemented with 0.5% glucose (GM17) at 30 °C without shaking or on GM17 plates containing 1.5% agar. The phages originated from disturbed milk fermentations produced using mixed starter cultures. Whey samples were collected over a period of 3 years from dairy plants in 8 different locations across Poland. Phage propagation was performed on *L*. *lactis* strains using GM17 medium supplemented with 10 mM calcium chloride (GM17-CaCl_2_), as described below. The obtained phage lysates were filtered and stored at 4 °C until further use.

### Preparation of phage lysates and phage DNA extraction

Phage lysates and DNA were obtained from 90 isolates deriving from industrial wheys based on known methods^52^. Briefly, phages were propagated on *L*. *lactis* IL1403 host strain grown to an optical density (OD_600_) of 0.15–0.2 and subsequently supplemented with 10 mM calcium chloride and infected with phage lysate from stock. The infected cell culture was further incubated at 30 °C or room temperature until lysis occurred. The obtained lysates were filtered using 0.45 µm filters (Stericup Millipore). RNase and DNase (Sigma) were added to the lysates to a final concentration of 20 µg ml^−1^ each and incubated at 37 °C for 1 h. Then, NaCl and polyethylene glycol (PEG6000) were added to a final concentration of 1 M and 10% (w/v), respectively, and after complete dissolution by brisk shaking, the lysates were stored overnight at 4 °C. Phage particle precipitates were concentrated by centrifugation at 9,000 × *g* for 20 min at 4 °C. The supernatant was discarded, and the phage pellet was resuspended in TM buffer (10 mM Tris-HCl, pH 8, 10 mM MgCl_2_). Chloroform (1:1, v/v) was added to the phage suspension and vortexed vigorously to extract PEG6000 and cell debris. The water phase containing phage particles was recovered after 15 min of centrifugation at 21,000 × *g* and 4 °C. Then, the following extractions were performed: once with phenol (1:1, v/v) and lithium chloride (1:10, v/v), twice with phenol alone (1:1, v/v) and once with phenol/chloroform solution (1:1, v/v). Subsequently, DNA was precipitated from the water phase with cold 96% isopropanol (1:2, v/v), concentrated by centrifugation at 21,000 × *g* for 30 min at 4 °C, and finally washed with 70% ethanol. The precipitates were then dried using the SpeedVac concentrator (Eppendorf) and dissolved in 100 μl TE buffer (10 mM Tris-HCl, 1 mM EDTA, pH 8) overnight at 4 °C, after which the DNA was ready for analysis.

### Restriction profiling of phage genomes

Phage DNA digestion and agarose gel electrophoresis were carried out as previously established^52^. Essentially, the genomic DNA of 90 phage isolates was cut separately with *EcoRI*, *EcoRV* or *HindIII* restriction endonucleases (Fermentas) overnight at 37 °C as recommended by the manufacturer. Enzymes were selected based on their ability to recognize and cleave within 6 nucleotide sites of low G-C content (33%), typical for lactococcal phages and their host genomes. The digested DNA fragments were separated on a 0.7% (w/v) agarose gel containing ethidium bromide in 1xTAE buffer (Merck). DNA loading dye with 50% formamide was used to load the samples on the agarose gel. Formamide was added to separate the phage cohesive (*cos*) ends^53^. Comparative analysis based on DNA restriction patterns allowed the separation of the phages into distinct restriction groups (8 in total). Representatives from each group were taken for sequencing.

### Host range determination

The infection specificity of the 8 sequenced phages was tested against a total of 60 *L*. *lactis* strains, including industrial^43^, laboratory^6^ and environmental^11^ isolates from the IBB PAS collection. Individual phage lystes (5 µl) were spotted separately on double-layer GM17-CaCl_2_ plates seeded with 200 µl of overnight-grown culture of selected bacterial strains, as described elsewhere, with minor modifications^50^. After overnight incubation at 30 °C, plates were examined for the presence of lytic zones, which were considered evidence of strain sensitivity.

### One-step growth assay

One-step growth studies were performed essentially as described by Chow *et al*.^54^. For this, the phage-sensitive *L*. *lactis* IL1403 indicator strain (10 ml) was grown in GM17 broth to OD_600_ ∼ 0.4. Then, the cell culture was centrifuged, and the pellet was resuspended in 2 ml of liquid GM17-CaCl_2_ and infected with the phage at a multiplicity of infection (MOI) of 0.01. After a 5-min incubation at 30 °C (adsorption of phages to bacterial cells), the culture was centrifuged (4,500 × *g*, 4 min), and the cell pellet was resuspended in 10 ml of GM17-CaCl_2_ and further incubated at 30 °C. Aliquots were taken at 3-min intervals until lysis of bacterial culture was complete. Serial dilutions of samples from each time point were immediately plated on the sensitive *L*. *lactis* host strain using the two-layer plates method, as described elsewhere^55^. The phage concentration was calculated as the number of PFU per ml, determined by counting the number of visible lysis zones after overnight incubation at 30 °C. The phage burst size was calculated by dividing the average final count of liberated phage particles (at plateau) by the initial average count of infected bacterial cells. The latent period was determined as the time period prior to the release of infection particles. Experiments were repeated in triplicate.

### Morphology analysis by electron microscopy

Electron microscopy analysis was performed for the 8 sequenced phages as described elsewhere^29^. Lysates of each phage were concentrated by centrifugation (20,000 × *g*, 4 °C for 4 h). Then, the pellet was washed with 100 mM ammonium acetate, centrifuged (20,000 × *g*, 4 °C for 1 h) three times and stained using phosphoric potassium tungstate (2% w/v) on a Formvar-coated copper grid. Electron micrographs were taken in the Laboratory of Electron Microscopy at the Nencki Institute of Experimental Biology (Warsaw, Poland) using the high-performance JEM 1400 transmission electron microscope (JEOL Co., Japan, 2008) equipped with an energy-dispersive full range X-ray microanalysis system (EDS INCA Energy TEM, Oxford Instruments, UK), tomographic holder and 11 megapixel MORADA G2 TEM Camera (EMSIS GmbH, Germany).

### Phage classification by multiplex PCR analysis

The confirmation of phage classification to 936-type phages was performed on phage lysates (1 µl) as templates using multiplex PCR with 936- (5′-TCAATGGAAGACCAAGCGGA-3′/5′-GTAGGAGACCAACCCAAGCC-3′) and c2-specific (5′-CAGGTGTAAAAGTTCGAGAACT-3′/5′-CAGATAATGCACCTGAATCA-3′) primers, as described earlier^56^. PCR products were separated on a 0.7% (w/v) agarose gel in 1xTAE buffer (Merck), stained with ethidium bromide, and visualized under UV light on a G:BOX instrument (Syngene).

### Genome sequencing and annotation

Sequencing of bacteriophage DNA samples was performed in the Laboratory of DNA Sequencing and Oligonucleotide Synthesis (IBB PAS) using Illumina Sequencing Technology. Open reading frames (ORFs) were automatically predicted using RAST (Rapid Annotation using Subsystem Technology) software. The BLASTp algorithm^57^ was run manually to assist in the functional annotation of detected *orfs* based on comparison with proteins in the non-redundant (nr) database of NCBI (http:// ftp.ncbi.nih.gov/blast/db).

### Bioinformatic analyses

Multiple sequence alignments were generated using MUSCLE (v3.7) as implemented within the Phylogeny.fr online platform (http://www.phylogeny.fr/)^58,59^. Functional sequence analyses of the putative phage products of unknown function were performed using the HMMER and HHPRED programs (https://toolkit.tuebingen.mpg.de/#/)^60,61^. The tRNA scanning SE and ARAGON software programs were used to search for tRNAs and tmRNAs^62,63^. Phage genomes were visualized using Easyfig. 2.1 software^64^.

### Phylogenetic studies

The evolutionary distance between the studied phages (or their respective proteins) was determined using the tools implemented in the Geneious® 8.1.8 program^65^. Multiple sequence alignments were generated using MUSCLE with 8 iterations (default settings). A maximum-likelihood tree was constructed using PHYML and the Junker-Castor substitution model with 100 bootstrap resamplings^66^.

### Data availability

The data generated during this study are disclosed in this article (and its Supplementary Materials file). Complete genomic sequences of the phages analysed in this study are deposited in GenBank under the given accession numbers with restricted access until publication of this article and are available from the corresponding author on reasonable request.

## Electronic supplementary material

Supplementary Materials
