# Supplementary material for: Molecular, physiological and phylogenetic traits of Lactococcus 936-type phages from distinct dairy environments
Source: Sci Rep. 2018 Aug 22;8:12540. doi: 10.1038/s41598-018-30371-3 (PMC6105707; doi:10.1038/s41598-018-30371-3)
Supplement: Supplementary file 1 — Supplementary Materials [file 41598_2018_30371_MOESM1_ESM.pdf]

Supplementary Figures

Molecular, physiological and phylogenetic traits of *Lactococcus* 936-type phages from distinct dairy environments

Chmielewska-Jeznach M, Bardowski JK, Szczepankowska AK

Supplementary Figure S1. Multiple sequence alignment of putative DNA polymerase subunits of *Lactococcus* phages sequenced in this study and phages bIBB29, R3.4 and P680 as the nearest homologues. Similar residues are coloured according to their conservation based on BLOSUM62 scoring (max: 3.0, light blue; low: 0.5, grey).

```
Eg1         MTSLFDKVSTAKELKESdDFAGGLL---WNVkDILPKGSLGLITGSEKSMKSSLAQDLAQ
bIBB29      MTSLFDKVSTAKELKESdDFAGGLL---WNVkDILPKGSLGLITGSEKSMKSSLAQDLAQ
5g1         MTSLFDKVSTAKELKESEDFSGGLL---WNVQDILPKGSLGLITGSEKSMKSSLAQDLAQ
24tp1       MTSLFDKVSTAKELKESEDFSGGLL---WNVQDILPKGSLGLITGSEKSMKSSLAQDLAQ
77s         MTSLFDKVSTAKELKESEDFSGGLL---WNVQDILPKGSLGLITGSEKSMKSSLAQDLAQ
14s         MTSLFDKVSTAKELKESEDFSGGLL---WNVQDILPKGSLGLITGSEKSMKSSLAQDLAQ
F14         MTniFnKVSTAKELKESEDFSGGLL---WNVQDILPKGSLGLITGSEKSMKSSLAQDLAQ
P680        MTniFeKVqTAKhLKErEDlin--LKDDWlidtlmPssqaGilvapfKSfKSSLAmhmAl
F12         MTniFnKVqTAKhLKErEDlin--LKDDWlidtlmPssqaGilvapfKSfKSSLAmhmAl
F13         MTniFnKVqTAKhLKErEDlin--LKDDWlidtlmPssqaGilvapfKSfKSSLAmhmAl
R3.4        MTniFDKVqTAKhLKErEDlin--LKDDWlidtlmPssqaGilvapfKSfKSSLAmhmAl

Eg1         AMALGEPFAGRETTKTNVLFIQNENSRLTEHQRLKGSRRDSPDNLYFLHGGAFKLDTWKY
bIBB29      AMALGEPFAGRETTKTNVLFIQNENSRLTEHQRLKGSRRDSPDNLYFLHGGAFKLDTWKY
5g1         AMALGEPFAGRETTKTNVLFIQNENSRLTEHQRLKGSRRDSPDNLYFLHGGAFKLDTWKY
24tp1       AMALGEPFAGRETTKTNVLFIQNENSRLTEHQRLKGSRRDSPDNLYFLHGGAFKLDTWKY
77s         AMALGEPFAGRETTKTNVLFIQNENSRLTEHQRLKGSRRDSPDNLYFLHGGAFKLDTWKY
14s         AMALGEPFAGRETTKTNVLFIQNENSRLTEHQRLKGSRRDSPDNLYFLHGGAFKLDTWKY
F14         AMALGEPFAGRETTKTNVLFIQNENSRLTEHQRLKGSRRDSPDNLYFLHGGAFKLDTWKY
P680        mvsqGlPFfGydTkrsktLyIdNEdtdrelnkRLr-nkdtaPedLhFLtGGeFmLD----
F12         mvsqGlPFfGydTkrsktLyIdNEdtdrelnkRLr-nkdtaPedLhFLtGGeFmLD----
F13         mvsqGlPFfGydTkrsktLyIdNEdtdrelnkRLr-nkdtaPedLhFLtGGeFmLD----
R3.4        mvsqGlPFfGydTkrsktLyIdNEdtdrelnkRLr-nkdtaPedLhFLtGGeFmLD----

Eg1         DSQGKKHNVGLRELYNFILEKDIGLVILDPLKDLLEDNDIINANQPMAEVLRGITNLRNT
bIBB29      DSQGKKHNVGLRELYNFILEKDIGLVILDPLKDLLEDNDIINANQPMAEVLRGITNLRNT
5g1         DSQGKKHNVGLRELYNFILEKDIGLVILDPLKDLLEDNDIINANQPMAEVLRGITNLRNT
24tp1       DSQGKKHNVGLRELYNFILEKDIGLVILDPLKDLLEDNDIINANQPMAEVLRGITNLRNT
77s         DSQGKKHNVGLRELYNFILEKDIGLVILDPLKDLLEDNDIINANQPMAEVLRGITNLRNT
14s         DSQGKKHNVGLRELYNFILEKDIGLVILDPLKDLLEDNDIINANQPMAEVLRGITNLRNT
F14         DSQGKKHNVGLRELYNFILEKDIGLVILDPLKDLLEDNDIINANQPMAEVLRGITNLRNT
P680        DS----HhmnL--LYeyIkEnDIkfVILDnLmtmLrngDIIygkd-fepmLRrITrLkll
F12         DS----HhmnL--LYeyIkdnDIkfVILDnLmtmLrngDIIygkd-fepmLRrITrLkll
F13         DS----HhmnL--LYeyIkdnDIkfVILDnLmtmLrngDIIygkd-fepmLRrITrLkll
R3.4        DS----HhmnL--LYeyIkEnDIkfVILDnLmtmLrngDIIygkd-fepmLRrITrLkll

Eg1         LDMKHDKYVTFMIVAHARKQAGEQSLTERDFRIIPSHILGATTIPsWYEiAFTMSPKINS
bIBB29      LDMKHDKYVTFMIVAHARKQAGEQSLTERDFRIIPSHILGATTIPsWYEiAFTMSPKINS
5g1         LDMKHDKYVTFMIVAHARKQsGEQSLTERDFRIIPSHILGATTIPsWYEiAFTMSPKINS
24tp1       LDMKHDKYVTFMIVAHARKQAGEQSLTERDFRIIPSHILGATTIPAWYEvAFTMSPKINS
77s         LDMKHDKYVTFMIVAHARKQAGEQSLTERDFRIIPSHILGATTIPAWYEvAFTMSPKINS
14s         LDMKHDKYVTFMIVAHARKQAGEQSLTERDFRIIPSHILGATTIPAWYEvAFTMSPKINS
F14         LDMKHDKYVTFMIVAHARKQAGEQSLTERDFRIIPSHILGATTIPAWYEvAFTMSPKINS
P680        f-----qdVTFllVAHAnKsAyanSmddkaymvkPSdaLGgsTltAWaEFmlmlSPK--r
F12         f-----qdVTFllVAHAnKsAyanSmddkaymvkPSdaLGgsTltAWaEFmlmlSPK--r
F13         f-----qdVTFllVAHAnKsAyanSmddkaymvkPSdaLGgsTltAWaEFmlmlSPK--r
```

```
R3.4            f-----qdVTFllVAHAnKsAyanSmddkaymvkPSdaLGgsTltAWaEFmlmlSPK--r

Eg1             KTKNrYSIMKVFARNFAFNDEILWGYVGSAFTSIEQDKKEPDSELVEKVKRETPIETTKE
bIBB29          KTKNrYSIMKVFARNFAFNDEILWGYVGSAFTSIEQDKKEPDSELVEKVKRETPIETTKE
5g1             KTKNrYSIMKVFARNFAFNnEILWGYVGSAFTSIEQDKKEPDSELVEKVKRETPIETTKE
24tp1           KTKNgYSIMKVFARNFAFNnEILWGYVGSvFTSIEQDKKEPDSELiEeVKqETPIETTKE
77s             KTKNgYSIMKVFARNFAFNnEILWGYVGSAFTSIEQDKKEPDSELVEKVKRETPIETTKE
14s             KTKNgYSIMKVFARNFAFNnEILWGYVGSAFTSIEQDKKEPDSELVEKVKRETPIETTKE
F14             KTKNgYSIMKVFARNFAFNnEILWGYVGSAFTSIEQDKKEPDSELVEKVKRETPIETTKE
P680            gkhNDfSklsVkARgyqFdDdlnfsYVdSvFTcvnksKKEPDSELiEeVKqETPIETTKE
F12             gkyNDfSklsVkARgyqFdDdlnfsYVdSvFTcvnksKKEPDSELVEKVKRETPIETTKE
F13             gkyNDfSklsVkARgyqFdDdlnfsYVdSvFTcvnksKKEPDSELVEKVKRETPIETTKE
R3.4            gkhNDfSklsVkARgyqFdDdlnfsYVdSvFTcvnksKKEPDnELVEKVKaETPIETTKE

Eg1             SAQAFLDLAKEQGKVIEND
bIBB29          SAQAFLDLAKEQGKVIEND
5g1             SAQAFLDLAKEQGKVIEND
24tp1           SAQAFLDLAKEQGKVIEND
77s             SAQAFLDLAKEQGKVIEND
14s             SAQAFLDLAKEQGKVIEND
F14             SAQAFLDLAKEQGKVIEND
P680            SAQAFLDLAKEQGKVtEND
F12             SAQAFLDLAKEQGKVIEND
F13             SAQAFLDLAKEQGKVIEND
R3.4            SAQAFLDLAKEQGKVIEND
```

Supplementary Figure S2. Identification of transmembrane domains of the putative holins of *Lactococcus* phages sequenced in this study. Prediction of transmembrane domains was performed using the TMHMM tool (Krogh et al., 2001).

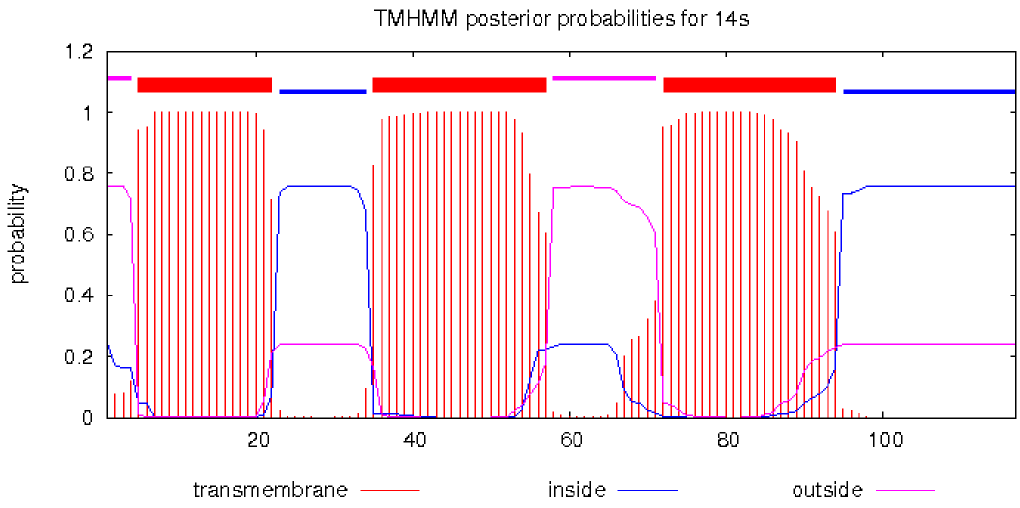

Supplementary Figure S3. Multiple sequence alignment of receptor binding proteins (RBPs) of *Lactococcus* phages sequenced in this study with protein representative of 936-type RBP subgroups recognized by Murphy et al. (2016). Similar residues are coloured according to their conservation based on BLOSUM62 scoring (max: 3.0, light blue; min: 0.5, grey).

```
645         MTIKkFTFFSPNGTEFPVsANaDAKLYMLLSEKDYSQFliRHWETPINTALNRIYTNTSL
340         MTIKkFTFFSPNGTEFPVsANaDAKLYMLLSEKDYSQFliRHWETPINTALNRIYTNTSL
P475        MTIKkFTFFSPNGTEFPVsANaDAKLYMLLSEKDYSQFslRHWETPINTALNRIYkNTSL
77s         MTIKNFTFFSPNGTEFPVGANNDAKLYMLLSEKDYSQFslRHWETPINTdLNRIYkNTSL
bIBB29      MTIKNFTFFSPNGTEFPVGANNDAKLYMLLSEKDYSQFslRHWETPINTALNRIYkNTSL
24tp1       MTIKNFTFFSPNGTEFPVGANNDAKLYMLLSEKDYSQFslRHWETPINTALNRIYkNTSL
5g1         MTIKNFTFFSPNGTEFPVGANNDAKLYMLLSEKDYSQFslRHWETPINTALNRIYkNTSL
F12         MTIKNFTFFSPNGTEFPVGANNDAKLYMLLSEKDYSQFslRHWETPINTALNRIYkNTSL
F13         MTIKNFTFFSPNGTEFPVGANNDAKLYMLLSEKDYSQFslRHWETPINTALNRIYkNTSL
F14         MTIKNFTFFSPNGTEFPVGANNDAKLYMLLSEKDYSQFslRHWETPINTALNRIYkNTSL
Eg1         MTIKNFTFFSPNGTEFPVGANNDAKLYMLLSEKDYSQFslRHWETPINTALNRIYkNTSL
14s         MTIKNFTFFSPNGTEFPVGANNDAKLYMLLSEKDYSQFslRHWETPINTALNRIYkNTSL
936         MTIKNFTFFSPNGTEFPVGsNNDAKLYMmLtgmDYktiRRkdWlTPtNsgLNvIYnNTSi
sk1         MTIKNFTFFSPNsTEFPVGsNNDgKLYMmLtgmDYrtiRRkdWssPlNTALNvqYTNTSi
p2          MTIKNFTFFSPNsTEFPVGsNNDgKLYMmLtgmDYrtiRRkdWssPlNTALNvqYTNTSi
712         MTIKNFTFFSPNGTEFPVGsNNDAKLYMmLtgmDYktiRRkdWvePlNTsLNvMYpNTSi
PhiC0139    MTIKNFTFFSPNGTEFPVGsNNDgKLYMmLtgmDYgtiRRkdWssPlNTALNvqYTNTSi
ASCC281     MTIKNFTFFSPNGTEFPVGsNNDgKLYMmLtgmDYgtiRRkdWssPlNiALNvqYTNTSi
P008        MTIKNFTFFSPNGTEFPVGsNNDgKLYMmLtgmDYgtiRRkdWtsPlNTALNvqYTNTSi
P113g       MTIKNFTFFSPNGTEFPVGsNNDgKLYMmLagmDYgtiRRkdWtsPlNTALNvqYTNTSi
bIL170      MTIKNFTFFSPNGTEFPVGsNNDgKLYMmLagmDYgtiRRkdWtsPlNTALNvqYTNTSi
PhiL.6      MTIKNFTFFSPNGTEFPVGsNNDgKLYMmLtgmDYgtiRRkdWtsPlNTALNvqYTNTSi
Phi155      MTIKyFTFFSPNnTEFPVGsNNDAKLYMmLtgmDYgtiRRkdWsaPVNTALNvqYTNTSi

645         LVGGRYFELYNEAVALNSNATNFVHANIDISNvTNPVTiSVETSnNSNkVDINTGVGVLK
340         LVGGRYFELYNEAVALNSNATNFVHANIDISNvTNPVTiSVETSnNSNkVDINTGVGVLK
P475        LVGGRYFELYdEAVALNSNATNFVHANIDISNaTNPVTmSVETSDNSNQVDINTGVGVLK
77s         LVGGRYFELYNEAVALNSNATNFVHANIDISNaTNPVTmSVETSDNSNQVDINTGVGVLK
bIBB29      LVGGRYFELYNEAVALNSNATNFVHANIDISNaTNPVTmSVETSDNSNQVDINTGVGVLK
24tp1       LVGGRYFELYNEAVALNSNATNFVHANIDISNaTNPVTmSVETSDNSNQVDINTGVGVLK
5g1         LVGGRYFELYNEAVALNSNATNFVHANIDISNaTNPVTmSVETSDNSNQVDINTGVGVLK
F12         LVGGRYFELYNEAVALNSNATNFVHANIDISNaTNPVTmSVETSDNSNQVDINTGVGVLK
F13         LVGGRYFELYNEAVALNSNATNFVHANIDISNaTNPVTmSVETSDNSNQVDINTGVGVLK
F14         LVGGRYFELYNEAVALNSNATNFVHANIDISNaTNPVTmSVETSDNSNQVDINTGVGVLK
Eg1         LVGGRYFELYNEAVALNSNATNFVHANIDISNaTNPVTmSVETSDNSNQVDINTGVGVLK
14s         LVGGRYFELYNEAVALNSNATNFVHANIDISNaTNPVTmSVETSDNSNQVDINTGVGVLK
936         iaGGRYFELsfEtVALNaNsvNyiHANIDltqTTrPVsLSaEkSDNSNsVDlNnaLGVLK
sk1         iaGGRYFELlNEtVALkgNAvNyiHANIDltrTaNPVsLSaETSnNSNgVDINnGsGVLK
p2          iaGGRYFELlNEtVALkgdsvNyiHANIDltqTaNPVsLSaETanNSNgVDINnGsGVLK
712         iaGGRYFELlsEtVALkaNsvNyiHANIDltkTaNPVsLSaEnvDNSNnVDfNnrsGVLK
PhiC0139    iaGGRYFELsNEtVALkpNsvNyiHANIDltqTThPVsLSaEnSnNSNnVDlNnnsGVLK
ASCC281     iaGGRYFELsNEtVALkpNsvNyiHANIDlSqTThPVsLSaETaDdSNnVDlNnnsGVLK
P008        iaGGRYFELsNEtVALkaNsvNyiHANIDltqTahPVTLSaETSnNSNnVDlNnnsGVLK
P113g       iaGGRYFELlNEtVALkgNAvNyiHANIDltqTahPVTLSaETSDNSNnVDlNnnsGVLK
bIL170      iaGGRYFELlNEtVALkgNAvNyiHANIDltqTahPVTLSaETSnNSNnVDlNnnsGVLK
PhiL.6      iaGGRYFELsNEtVALkgNsvNyiHANIDltqTahPVsLSaETinNSNhVDlNnssGVLK
```

```
Phi155      iaGGRYFELvgEtipLkaNsvNyiHANIDltqTTsPVkLSaETlnNSNrtDINnssGVLK

645         KCIEVVETNAiGVSAVTKPSGSSILEKIYP----VGAYYfSSQPTEPATLF-----GFGT
340         KCIEVVETNAiGVSAVTKPSGSSILEKIYP----VGAYYfSSQPTEPATLF-----GFGT
P475        KCIEVVETNAMGVSAVTKPSGSSIiEKIYP----VGAYYLSSQPTEPATLF-----GFGT
77s         KCIEVVETNAMGVSAVTKPSGSSIiEKIYP----VGAYYLSSQPTEPATLF-----GFGT
bIBB29      KCIEVVETNAMGVSAVTKPSGSSIiEKIYP----VGAYYLSSQPTEPATLF-----GFGT
24tp1       KCIEVVETNAMGVSAVTKPSGSSIiEKIYP----VGAYYLSSQPTEPATLF-----GFGT
5g1         KCIEVVETNAMGVSAVTKPSGSSIiEKIYP----VGAYYLSSQPTEPATLF-----GFGT
F12         KCIEVVETNAMGVSAVTKPSGSSIiEKIYP----VGAYYLSSQPTEPATLF-----GFGT
F13         KCIEVVETNAMGVSAVTKPSGSSIiEKIYP----VGAYYLSSQPTEPATLF-----GFGT
F14         KCIEVVETNAMGVSAVTKPSGSSIiEKIYP----VGAYYLSSQPTEPATLF-----GFGT
Eg1         KCIEVVETNAMGVSAVTKPSGSSIiEKIYP----VGAYYLSSQPTEPATLF-----GFGT
14s         KCIEVVETNAMGVSAVTKPSGSSIiEKIYP----VGAYYLSSQPTEPATLF-----GFGT
936         vlIdirtTNgtdVTlqkaPdnvtyLdKav-----InsiisSStvdsgqvktAtinTGhGr
sk1         vCfdiVtTsgtGVTstkptvqTStLdsIs-----VndmtvSgsidvPvqtl-tveAGnGl
p2          vCfdiVtTsgtGVTstkpivqTStLdsIs-----VndmtvSgsidvPvqtl-tveAGnGl
712         vlIdirtTdgMGVisekaPdnityLdKVv-----InsisttSgvlDi---------GnGv
PhiC0139    vvIdirtTNgiGVisdktPdnvtyLdKVi-----tnsleMkgfadsyisIF----StaGd
ASCC281     vvIdirtTNgtGVintktPdnvtyLdKVi-----tnsleMkgfadsyvafFANk--GgGn
P008        vvIdirtTNgtGVinaksPtehtLLddVi-----Ins--LvnkkdiPwTdl-NrASGvGs
P113g       vvIdirtTNgtGVinakQPtehtLLddVi-----Ins--LvnQkdvPwTdl-NrAgGvGs
bIL170      vvIdirtTNgtGVisaeQPtehtLLddVi-----Ins--LvnkkdiPwTdl-NrASGvGs
PhiL.6      vlIdirtTNgLGVisseiPkqittLdelatKTAkIedltvkgdikgwtsVs----mqnva
Phi155      vlIdirtTNgLGVisseiPkqittLdelatKTAnIedltvkgdikgwtsVs----mqnva

645         WSRVKGRGLvGVD-ESDSAlSsGGKQG-----GS-----TNPLSQHTITPSSGQFVVARG
340         WSRVKGRGLvGVD-ESDSAlSsGGKQG-----GS-----TNPLSQHTITPSSGQFVVARG
P475        WSRVKGRGLvGVD-ESDSAlSsGGKQG-----GS-----TNPLSQHTITPSSGQFVVARG
77s         WSRVKGRGLvGVD-ESDSAlSsGGKQG-----GS-----TNPLSQHTITPSSGQFVVARG
bIBB29      WSRVKGRGLvGVD-ESDSAlSsGGKQG-----GS-----TNPLSQHTITPSSGQFVVARG
24tp1       WSRVKGRGLvGVD-ESDSAlSsGGKQG-----GS-----TNPLSQHTITPSSGQFVVARG
5g1         WSRVKGRGLvGVD-ESDSAlSsGGKQG-----GS-----TNPLSQHTITPSSGQFVVARG
F12         WSRVKGRGLvGVD-ESDSAlSsGGKQG-----GS-----TNPLSQHTITPSSGQFVVARG
F13         WSRVKGRGLvGVD-ESDSAlSsGGKQG-----GS-----TNPLSQHTITPSSGQFVVARG
F14         WSRVKGRGLvGVD-ESDSAlSsGGKQG-----GS-----TNPLSQHTITPSSGQFVVARG
Eg1         WSRVKGRGLvGVD-ESDSAlSsGGKQG-----GS-----TNPLSQHTITPSSGQFVVARG
14s         WSRVKGRGLvGVD-ESDSAlSsGGKQG-----GS-----TNPLSQHTITPSSGQFVVARG
936         sAvItriGntvti-tSqSqySsepanG-----sw-----ergvStlpVg-----FrpAte
sk1         dlQltkknndlVi--vrffgSvsniQk-----GwNM---sgtwvdrpfrPavvQsLV---
p2          qlQltkknndlVi--vrffgSvsniQk-----GwNM---sgtwvdrpfrPaAvQsLV---
712         tfswq-kkadiVE------fhwGGRltsins-GAsfpvkapfqlipdkvkelv-------
PhiC0139    titItapwdctaE--vElfyhgwGygGGeWEIG--------------IngSNGvtktyei
ASCC281     tvtftapwdctaE-velfyhgwG-ygGGeWEIGi-----TtPsgltqIyeaT--------
P008        tgilqaRiInsViYvrgNsipvpnvap-----nf-----ivPvg--TfpPafGtnLpqfd
P113g       tgtlqaRiInGViYvrgNsipvpnvap-----nf-----ivPvg--TfpPafGtnLpqfd
bIL170      tgilqaRiInGViYvrgNsipvpnvap-----nf-----ivPvg--TfpPafGtnLpqfd
PhiL.6      sAtlqykkInGVi-clrgsgnwGafka-----nS-----Tkvvg--sLpPevrptdntqf
Phi155      sAtlqykkInGai-clrgsgnwGafka-----nS-----Tkvvg--sLpPevrptdntqf

645         AGnrHWSSGGAPSNSYAMDTENGGV--TVGDNTNHNNWQPFEAAYIWKRIN----------
340         AGnrHWSSGGAPSNSYAMDTENGGV--TVGDNTNHNNWQPFEAAYIWKRIN----------
P475        AGnrHWSSGGAPSNSYAMDTENGGV--iVGDNTNHNNWQPFEAAYIWKRIN----------
77s         AGKWHWSSGGAPSNSYAMDTENGGI--TVGDNTNHNNWQPFEAAYIWKRIN----------
bIBB29      AGKWHWSSGGAPSNSYAMDTENGGV--TVGDNTNHNNWQPFEAAYIWKRIN----------
24tp1       AGKWHWSSGGAPSNSYAMDTENGGV--TVGDNTNHNNWQPFEAAYIWKRIN----------
5g1         AGKWHWSSGGAPSNSYAMDTENGGV--TVGDNTNHNNWQPFEAAYIWKRIN----------
```

```
F12             AGKWHWSSGGAPSNSYAMDTENGGI--TVGDNTNHNNWQPFEAAYIWKRIN----------
F13             AGKWHWSSGGAPSNSYAMDTENGGI--TVGDNTNHNNWQPFEAAYIWKRIN----------
F14             AGKWHWSSGGAPSNSYAMDTENGGI--TVGDNTNHNNWQPFEAAYIWKRIN----------
Eg1             AGKWHWSSGGAPSNSYAMDTENGGI--TVGDNTNHNNWQPFEAAYIWKRIN----------
14s             AGKWHWSSGGAPSNSYAMDTENGGI--TVGDNTNHNNWQPFEAAYIWKRIN----------
936             tliYnhdltipskfSwnllhtNGlI-----DlfsngN--ikttdYIltsgqFwiTkdkLpe
sk1             ---gHfAg---rdtSFhiDinpnGsitwwGEsidntp--iatrgngsyfIk----------
p2              ---gHfAg---rdtSFhiDinpnGsitwwGaNidktp--iatrgngsyfIk----------
712             ---gHfpt---tSNSFhiDlEhdGtfrwwGEdkas----gsvrgtamyfIk----------
PhiC0139        t--gy--tkGhdSqaiAMpTsaGftglkkGqqytfNk--rdvsgraggatrvkiiVkLyrN
ASCC281         ---gytNghnSqalampakaiysGL--kkGqqytfdk--rdasglaggskhemmiVkLykN
P008            ta-gtfyShGnlSlSl-inmspsGI--aVGn---------PnntsmngKtIsFvLSVPLL--
P113g           ta-gtfySpGhlSlSl-inmspsGI--aVGn---------PnntsmngKtIsFaLSaPLL--
bIL170          ss-gtfyShGnlSlSl-inmspsGI--aVGn---------PnntsmngKtIsFaLSaPLL--
PhiL.6          emttqrAnnnnkpmelliDTngtis--vwsysags----gnyggvVgtyfq----------
Phi155          emttqrAnnnnkpmelqiDTngtis--vwsysags----gnyggvVgtyfq----------
```

Supplementary Figure S4. Maximum-likelihood phylogenetic tree of receptor binding proteins (RBPs) of *Lactococcus* phages sequenced in this study. RBP phylogenies were reconstructed from multiple alignment of complete protein sequences, which were generated using MUSCLE with 8 iterations (default settings). A maximum-likelihood tree was constructed using PHYML and the Junker-Castor substitution model with 100 bootstrap resamplings. The scale bar represents evolutionary distance. Consensus support (%) is marked at each node.

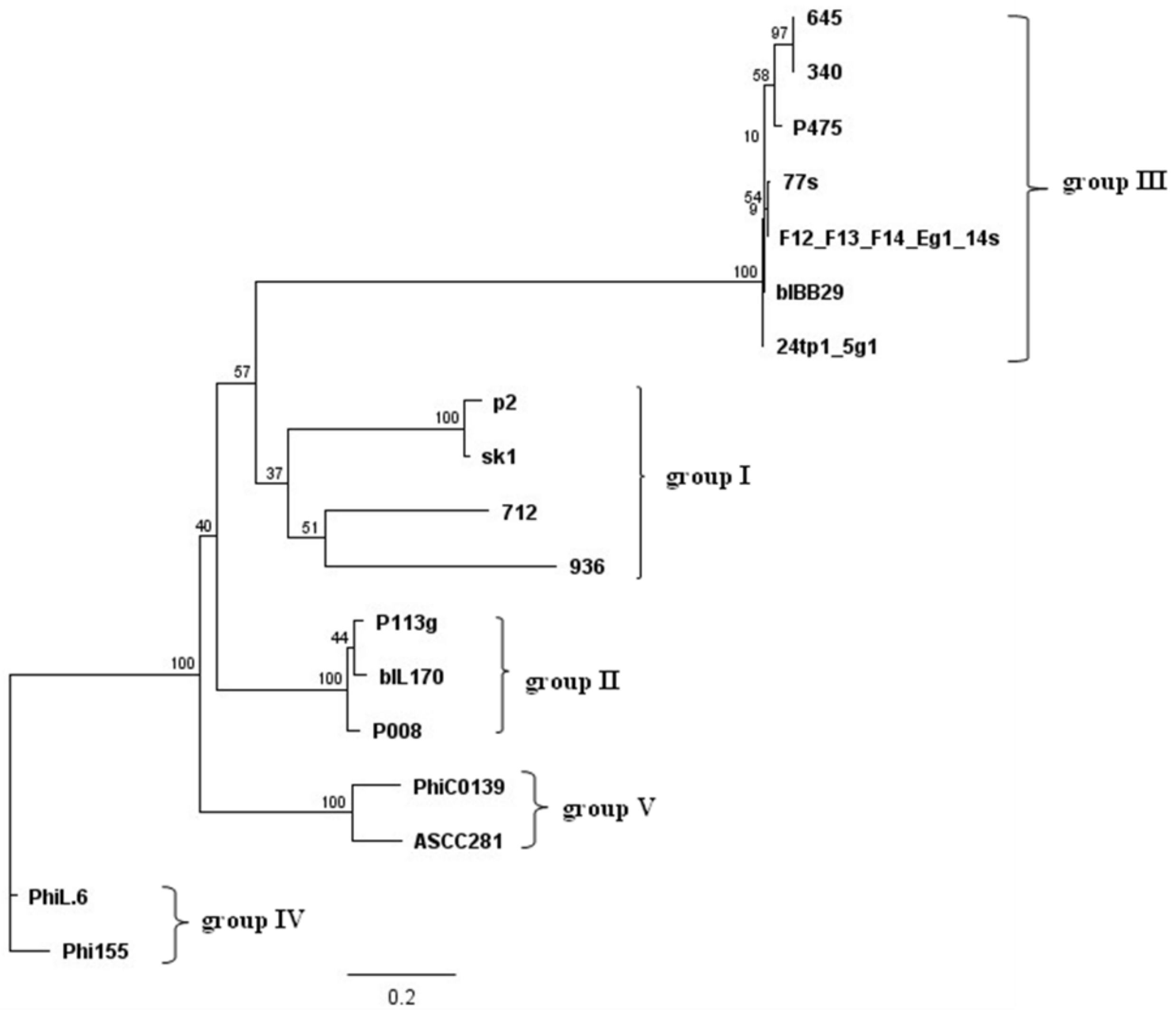

Supplementary Figure S5. Multiple sequence alignment of putative TpeX proteins of *Lactococcus* phages sequenced in this study with the TpeX identified in other 936-type phages (Murphy et al., 2016). Similar residues are coloured according to their conservation based on BLOSUM62 scoring (max: 3.0, light blue; min: 0.5, grey).

```
Phi145      -----mLNf----------SsgvtfGkhygwpagtatpsKnraysqkvkvtpndqytisA
Phi93       -----mLNf----------SsgvtfGkhygwpagtatpsKnraysqkvkvtpndqytisA
Eg1         MTVYPNLNLLKNTRTTSATSTSSAWGTLFSSEQIYDSAIKSKTGVSAMTLGFDVSVPLNA
24tp1       MTVYPNLNLLKNTRTTSATSTSSAWGTLFSSEQIYDSAIKSKTGVSAMTLaFDVSVPLNA
F14         MTVYPNLNLLKNTRTTSATSTSSAWGTLFSSEQIYDSAIKSKTGVSAMTLGFDVSVPLNA
5g1         MTVYPNLNLLKNTRTTSATSTSSAWGTLFSSEQIYDSAIKSKTGVSAMTLGFDVSVPLNA
F12         MTVYPNLNLLKNTRTTSATSTSSAWGTLFSSEQIYDSAIKSKTGVSAMTLGFDVSVPLNA
F13         MTVYPNLNLLKNTRTTSATSTSSAWGTLFSSEQIYDSAIKSKTGVSAMTLGFDVSVPLNA
77s         -------NLLKNTRTTSATSTSSAWGTLFSSEQIYDSAIKSKTGVSAMTLGFDVSVPLNA
14s         MTVYPNLNLLKNTRTTSATSTSSAWGTLFSSEQIYDSAIKSKTGVSAMTLGFDVSVPLNA

Phi145      dwkNanwlsiyGftnegdttavAr-ygsanGtGTGsWdsnfvggytaandmtftVpdnvn
Phi93       dwkNanwlsiyGftnegdttavAr-ygsanGtGTGsWdsnfvggytaandmtftVpdnvn
Eg1         KVGNNISVQLKGQNSQAHGNVGANDFNTIVGE---KWHNIEQSDLGKkIRLSTSVELDPK
24tp1       KVGNNISVQLKGQNSQAHGNVGANDFNTIVGE---KWHNIEQSDLGKTIRLSTSVELDPK
F14         KVGNNISVQLKGQNSQAHGNVGANDFNTIVGE---KWHNIEQSDLGKTIRLSTSVELDPK
5g1         KVGNNISVQLKGQNSQAHGNVGANDFNTIVGE---KWHNIEQSDLGKTIRLSTSVELDPK
F12         KVGNNISVQLKGQNSQAHGNVGANDFNTIVGE---KWHNIEQSDLGKTIRLSTSVELDPK
F13         KVGNNISVQLKGQNSQAHGNVGANDFNTIVGE---KWHNIEQSDLGKTIRLSTSVELDPK
77s         KVGNNISVQLKGQNSQAHGNVGANDFNTIVGE---KWHNIEQSDLGKTIRLSTSVELDPK
14s         KVGNNISVQLKGQNSQAHGNVGANDFNTIVGE---KWHNIEQSDLGKTIRLSTSVELDPK

Phi145      YivasyAskspDtlTlq------GLlngKpKLEqGSkvTPWmrSSSEtiesDYPSYIGnY
Phi93       YivasyAskspDtlTlq------GLlngKpKLEqGSkvTPWmrSSSEtiesDYPSYIGnY
Eg1         YqSFDTALADTDSITIRQVEGTPGLVYSKLKLEPGSTDTPWIPSSSEVTAEDYPSYIGTY
24tp1       YqSFDTALADTDSITIRQVEGTPGLVYSKLKLEPGSTDTPWIPSSSEVTAEDYPSYIGTY
F14         YHSFDTALADTDSITIRQVEGTPGLVYSKLKLEPGSTDTPWIPSSSEVTAEDYPSYIGTY
5g1         YHSFDTALADTDSITIRQVEGTPGLVYSKLKLEPGSTDTPWIPSSSEVTAEDYPSYIGTY
F12         YHSFDTALADTDSITIRQVEGTPGLVYSKLKLEPGSTDTPWIPSSSEVTAEDYPSYIGTY
F13         YHSFDTALADTDSITIRQVEGTPGLVYSKLKLEPGSTDTPWIPSSSEVTAEDYPSYIGTY
77s         YHSFDTALADTDSITIRQVEGTPGLVYSKLKLEPGSTDTPWIPSSSEVTAEDYPSYIGTY
14s         YHSFDTALADTDSITIRQVEGTPGLVYSKLKLEPGSTDTPWIPSSSEVTAEDYPSYIGTY

Phi145      TgKivdgQSTDPvrYnWKKIE
Phi93       TgKivdgQSTDPvrYnWKKIE
Eg1         TDKNSNEQSTDPEKYTWKKIE
24tp1       TDKNSNEQSTDPEKYTWKKIE
F14         TDKNSNEQSTDPEKYTWKKIE
5g1         TDKNSNEQSTDPEKYTWKKIE
F12         TDKNSNEQSTDPEKYTWKKIE
F13         TDKNSNEQSTDPEKYTWKKIE
77s         TDKNSNEQSTDPEKYTWKKIE
14s         TDKNSNEQSTDPEKYTWKKIE
```

Supplementary Figure S6. Maximum-likelihood phylogenetic tree of MTP extension of *Lactococcus* phages sequenced in this study with the MTP extensions identified in other 936-type phages (Murphy et al., 2016). MTP extension phylogenies were reconstructed from multiple alignment of major tail protein extension sequences generated using MUSCLE with 8 iterations (default settings). A maximum-likelihood tree was built using PHYML and the Junker-Castor substitution model with 100 bootstrap resamplings. The scale bar represents evolutionary distance. Consensus support (%) is marked at each node.

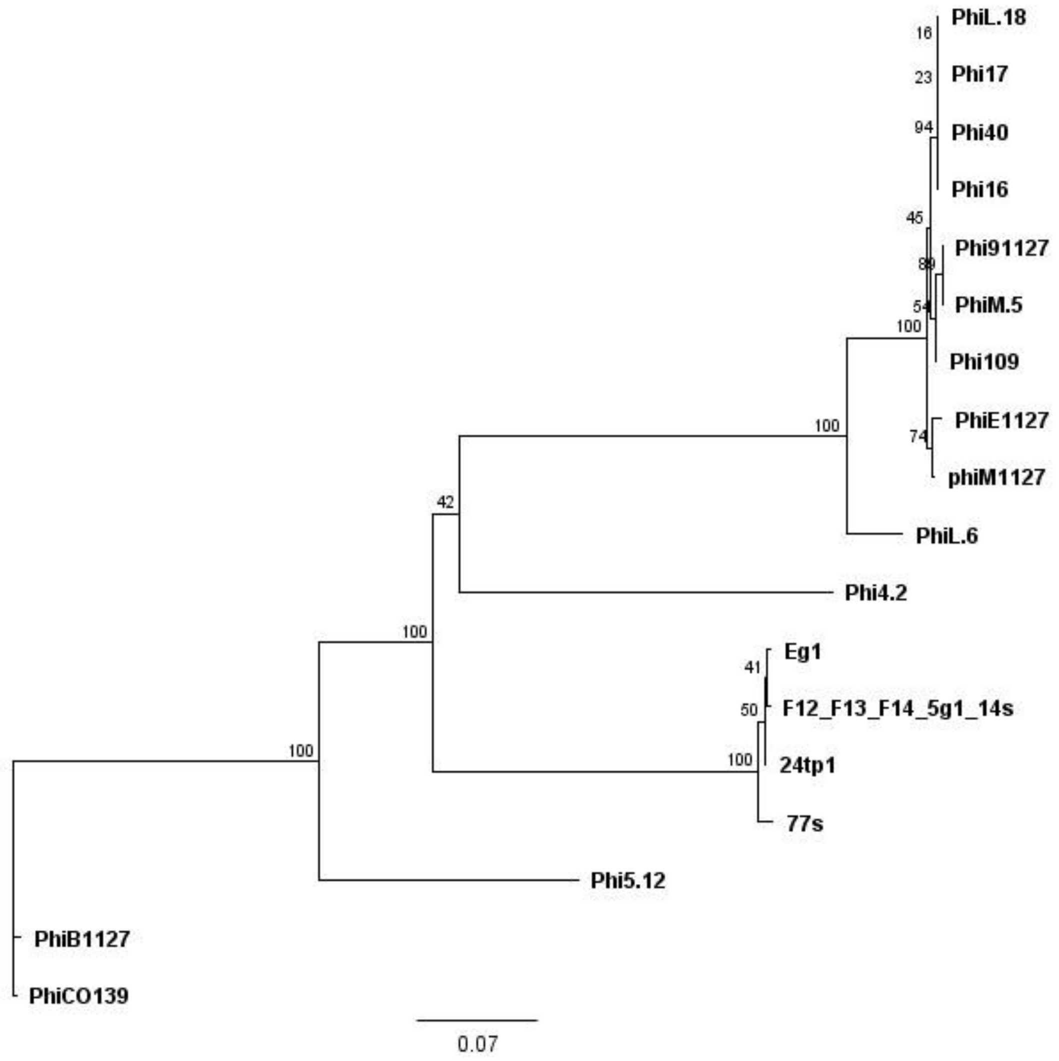

References

Krogh, A., Larsson, B., von Heijne, G. & Sonnhammer, E. L. L. Predicting transmembrane protein topology with a hidden Markov model: Application to complete genomes. *J. Mol. Biol.* **305**, 567-580 (2001).

Murphy, J. et al. Comparative genomics and functional analysis of the 936 group of lactococcal *Siphoviridae* phages. *Sci. Rep*. **6**, 21345 (2016).

Supplementary Tables.

Supplementary Table S1. Host range studies of *Lactococcus* phages sequenced in this study tested on industrial, laboratory and environmental *L. lactis* strains. Grey-shaded boxes indicate clear plaques; light grey boxes indicate turbid plaques formed on the specified strains.

	Bacteriophages							
	Eg1	F12	F13	F14	5g1	14s	77s	24tp1
**Industrial strains**								
L. lactis subsp. *cremoris*								
IBB694								
IBB695								
IBB696								
IBB697								
IBB698								
IBB699								
IBB700								
IBB1788								
IBB1789								
IBB1790								
IBB1791								
IBB1792								
L. lactis subsp. *lactis*								
IBB743								
IBB749*								
IBB750*								
IBB752								
IBB753								
IBB754								
IBB757								
IBB758								
IBB759								
IBB761								
IBB763								
IBB764								
IBB1784								
IBB1796								
L. lactis subsp. *lactis* biovar. *diacetylactis*								

IBB734*
IBB735*
IBB736*
IBB737*
IBB738*
IBB742
IBB744
IBB745
IBB746
IBB747
IBB748*
IBB751*
IBB755
IBB756
IBB760
IBB762
IBB1286*
**Natural isolates**
L. lactis subsp. *cremoris*
IBB338
IBB339
IBB340
IBB341
IBB342
IBB704
L. lactis subsp. *lactis*
10LC
other *Lactococcus* species
L. garvieae 3LC
L. plantarum 76B
L. laudensis 115
L. raffinolactis 87B
**Laboratory strains**
L. lactis subsp. *cremoris*
LM0230
MG1363
IBB477
NZ9000
L. lactis subsp. *lactis* biovar. *diacetylactis*
IL1403

F7/2			

*The industrial starter strains used at the time of whey sample collection.
